# Artificial Intelligence Across the Drug Development Lifecycle

**DOI:** 10.3390/medsci14020248

**Published:** 2026-05-10

**Authors:** Grigory Demyashkin, Mikhail Parshenkov, Sergey Zyryanov, Alexander Yavorskiy, Petr Shegai, Andrey Kaprin

**Affiliations:** 1 Department of Digital Oncomorphology, National Medical Research Centre of Radiology, 2nd Botkinsky Pass., 3, 125284 Moscow, Russia; 2 Department of Industrial Pharmacy, Institute of Professional Education of the I.M. Sechenov First Moscow State Medical University of the Russian Ministry of Health (Sechenov University), Trubetskaya st., 8/2, 119048 Moscow, Russia; 3 Department of General and Clinical Pharmacology, Peoples’ Friendship University of Russia (RUDN University), Miklukho-Maklaya st., 6, 117198 Moscow, Russia; 4 Autonomous Non-Profit Organization Association of Participants in the Circulation of Medicines and Medical Devices “Lekmedobrashchenie”, 119333 Moscow, Russia; 5 Department of Urology and Operative Nephrology, Peoples’ Friendship University of Russia (RUDN University), Miklukho-Maklaya st., 6, 117198 Moscow, Russia

**Keywords:** artificial intelligence, machine learning, drug development, drug discovery, clinical trials, nonclinical trials, precision medicine

## Abstract

Artificial intelligence (AI) is becoming a central driver of change across the drug development lifecycle. However, its integration is evolving so rapidly that it remains essential to understand how these technologies are currently positioned within the field. Because reliable access to high-quality (effective and safe) drugs is essential to public health, the pharmaceutical product lifecycle (PPL) offers a coherent framework for evaluating how AI can enhance evidence and data creation across all stages. To understand where AI genuinely adds value, this review examines its contribution across the major stages of the PPL. Rather than treating drug discovery, nonclinical evaluation, clinical research, and post-marketing assessment as separate domains, we view them as a continuous chain of data, where digital technologies enhance different decision points in distinct ways. In early discovery, AI narrows the search space by integrating diverse datasets to prioritize candidates most likely to succeed. Nonclinical models increasingly rely on machine-learning systems designed to improve the human relevance of safety predictions. Within clinical trials, AI supports cohort formation, real-time monitoring, and new analytic strategies that supplement empirical evidence. Case studies from leading pharmaceutical companies illustrate that the most meaningful advances emerge when AI is embedded not as a standalone tool but as part of a broader data strategy that links information across stages. Taken together, current evidence suggests that AI is beginning to transform data generation and integration throughout the PPL. Given the accelerating pace of digital innovation, it is essential for the field to maintain continuous awareness of emerging methodologies and evolving regulatory frameworks to ensure that these technologies are implemented in a reliable, transparent, and scientifically grounded manner.

## 1. Introduction

Artificial intelligence (AI) has swiftly developed into a transformative force across numerous sectors, with its impact on healthcare and drug development proving particularly profound. The ability of AI algorithms to process vast, complex datasets, identify complex patterns, generate predictive insights, and reconstruct biological relationships across heterogeneous data sources has positioned it as a critical enabler for accelerating innovation within the pharmaceutical industry [1,2].

Global investments in healthcare AI underscore this burgeoning significance; for instance, during 2024, healthcare AI startups attracted close to $10.5 billion in funding distributed through 511 deals. Furthermore, by 2025, the proportion of health-tech capital flowing to AI firms increased to 55%, up from 37% in 2024. The global AI in healthcare market, valued at approximately $39 billion in 2025, is projected to reach $50 billion in 2026, with some estimates suggesting a substantial growth to over $1 trillion by 2034 [3,4,5]. The rapid market expansion highlights the recognized potential of AI to revolutionize drug development, making it an indispensable component of modern pharmaceutical strategy.

Despite the undeniable progress and significant investment, the comprehensive and systemic integration of digital technologies, particularly AI, across all stages of the drug lifecycle remains a complex and often fragmented endeavor. While AI has demonstrated remarkable successes in specific areas, for example, in nonclinical discovery, its seamless adoption throughout the entire continuum is far from complete [6,7]. In fact, this fragmentation is broader than it might seem at first glance. Andrew Bate and Philip Michael Tregunno raised a direct concern—the current literature on AI in the pharmaceutical industry (specifically pharmacovigilance) remains largely “task-specific”. Each group solves its own problem and publishes its own result but rarely asks how that result connects to decisions made before or after it in the development pipeline [8]. The “unknown” lies in understanding the intricate interplay of technical, regulatory, and organizational challenges that impede this holistic integration. Issues (e.g., heterogeneity of data, the “black box” nature of advanced AI models, the lack of standardized interoperability across diverse data sources) continue to pose significant hurdles, often leading to isolated AI applications rather than a fully integrated digital ecosystem [9,10].

Existing research has shed light on many ways AI can support drug development, often by examining a single method or focusing on one stage of the lifecycle. Studies of specific AI applications (e.g., generative chemistry, predictive toxicology, nonclinical data adoption, clinical trial design) have each demonstrated clear advances in their respective areas [11,12,13,14]. What is discussed less often is how these individual applications fit together. Because they are usually presented separately, it can be difficult to see how AI contributes across the full set of decisions made from discovery through post-marketing evaluation. A more integrated view can help clarify where current methods align, where gaps remain, and how AI may support the broader development process as it continues to evolve.

This article aims to bridge this gap by providing an “umbrella” examination of AI’s integration across the entire drug development lifecycle. We seek to move beyond isolated case studies to discuss the overarching trends, systemic challenges, and strategic opportunities presented by AI from initial discovery to market access and beyond. By drawing on recent academic studies and industry analyses, our review outlines the current use of AI across the major stages of drug development and describes emerging directions that may shape how AI is used in the future. Together, these elements provide a “360-view” of how AI is being incorporated into drug development today and what questions remain for researchers, industry leaders, and the wider scientific community.

## 2. Artificial Intelligence: The Epistemological Frontier in Drug Development

Over the past two decades, healthcare systems and the pharmaceutical industry have fundamentally reshaped the scientific and operational framework of drug development [15]. This reflects a broader transition from predominantly manual and sequential research processes toward data-rich (a term often described in the medical literature as “data-driven”), interconnected, and increasingly automated workflows that mirror the technological trajectory described across the “Industry 4.0 framework” conception [16,17,18].

The cost structure of drug development has shifted markedly: for example, between 2008 and 2024, the capitalized cost per approved therapeutic increased by 123 percent (from approximately $1 billion to $2.23 billion), outpacing general inflation by a factor of three, while mean development timelines extended by 40 percent, from 7.2 years to 10–15 years [19,20,21]. As these economic pressures accumulate, the imperative to develop new therapeutics intensifies the scientific and operational demands placed on drug development [22,23,24]. This increases the importance of methods capable of producing more consistent, efficient, and interpretable data at key stages of drug development. Against this backdrop, AI integration represents a strategic response.

Before examining specific applications within the PPL framework, we provide a brief technical overview of the computational methods referenced throughout this review. In the context of the following work, this “preliminary” foundation is necessary because the biomedical literature increasingly publishes results obtained using AI without specifying the class of algorithm on which they are based or the underlying validation logic. The following subsections provide definitions of the main families of methods; readers already familiar with machine learning (ML) and deep learning (DL) methodology may proceed directly to Section 2.1.

AI, in its broadest sense, embodies computational systems engineered to perform tasks that traditionally necessitate cognitive faculties, often achieving or exceeding agent-level performance within defined environments [25,26].

Mathematically, AI systems are fundamentally designed to identify optimal solutions or approximate complex functions:

∫:χ→γ,

where ***χ*** represents the input space and ***γ*** the output space. This objective is typically achieved through the minimization of an objective function:

$$J\left(\theta \right),$$

parameterized by ***θ***, which quantifies the discrepancy between the system’s output and a desired outcome. The process often involves iterative optimization algorithms that navigate a high-dimensional parameter space to converge towards a global or local minimum of ***J***(***θ***) [27,28].

Data provide the essential foundation for AI, with its quality, volume, and representativeness directly impacting model generalization and robustness. The principles governing data acquisition, preprocessing, and feature engineering are critical, as biases or inconsistencies in the input data can propagate through the algorithmic pipeline, leading to suboptimal or erroneous system predictions [29,30]. For this reason, rigorous attention to data quality (e.g., statistical distributions, dimensionality reduction, noise modeling) is essential for developing reliable and effective AI solutions [31].

Building on these principles that guide the development of effective AI solutions, machine learning (ML) refers to the subset of AI in which systems improve their performance on a task (T) through experience (E), as evaluated by a performance metric (P) [32,33]. Mathematically, ML is rooted in statistical learning theory, aiming to infer underlying patterns and relationships from observed data. This involves constructing a model ***M*** that can generalize from a finite set of training examples:

$$D=\left\{\left(x_{i},y_{i}\right)\right\}\begin{array}{c} N\\ i=1\; \end{array}$$

to make accurate predictions on unseen data [34]. The core challenge lies in minimizing the expected generalization error:

$$R\left(f\right)=E\left[L\left(Y,f\left(X\right)\right)\right],$$

where ***L*** is a loss function, by finding a function ***f*** within a hypothesis space that best approximates the true data-generating process. This is often achieved by minimizing the empirical risk:

$$R_{emp}\;(f)=\frac{1}{N}\sum_{i=1}^{N}L\left(y_{i},f\left(x_{i}\right)\right),$$

which serves as a proxy for the generalization error [35]. In pharmaceutical practice, the training set D typically consists of experimentally measured molecular data (e.g., binding affinities, solubility values, cytotoxicity endpoints, and others) paired with chemical structure representations [36,37]. The generalization error *R*(*f*) then quantifies the model’s ability to predict these properties for compounds it has never seen—the central question in any virtual screening or ADMET prediction test.

ML paradigms are broadly categorized by the nature of the feedback received during training (supervised learning, where models learn from labeled datasets; unsupervised learning, which identifies hidden structures in unlabeled data; and reinforcement learning, where an agent learns optimal policies through trial-and-error interactions with an environment, maximizing a cumulative reward signal) [38,39,40]. Each paradigm employs distinct algorithmic approaches, from linear regression and support vector machines to clustering algorithms and Q-learning, all underpinned by principles of optimization and statistical inference to extract actionable insights from data [41].

Building on the foundational principles of machine learning, Deep learning (DL) constitutes a specialized subfield that employs Artificial neural networks (ANNs) with multiple hidden layers to learn hierarchical feature representations directly from raw data [42]. The fundamental computational unit of an ANN is the neuron, which computes a weighted linear combination of its inputs:

$$z=\sum_{j=1}^{M}w_{j}x_{j}+b,$$

where $x_{j}$ denote input features, $w_{j}$ their corresponding weights, and *b* a bias term. This pre-activation value is then passed through a non-linear activation function

$$a=\sigma \left(z\right),$$

to produce the neuron’s output [43]. Common activation functions like the “Rectified linear unit” (ReLU):

$$ReLU\left(z\right)=max\left(0,z\right),$$

and the sigmoid function,

$$\sigma \left(z\right)=\frac{1}{1+e^{-z}},$$

introduce essential non-linearity that enables ANNs to model complex, non-linear relationships within data [44,45].

The training of deep architectures relies primarily on the backpropagation algorithm, an efficient procedure for computing the gradient of the loss function with respect to each weight and bias in the network [46]. Backpropagation applies the chain rule of calculus to propagate error gradients backward through the layers, enabling iterative parameter updates via gradient descent or its variants (e.g., Adam, RMSprop) to minimize empirical risk [47]. The considerable depth of modern DL models (frequently spanning tens or even hundreds of layers) facilitates the automatic extraction of increasingly abstract, hierarchical, and invariant features, making DL particularly powerful for high-dimensional modalities such as image, audio, and sequential data, which are particularly valuable in drug development [48].

**Figure 1:**
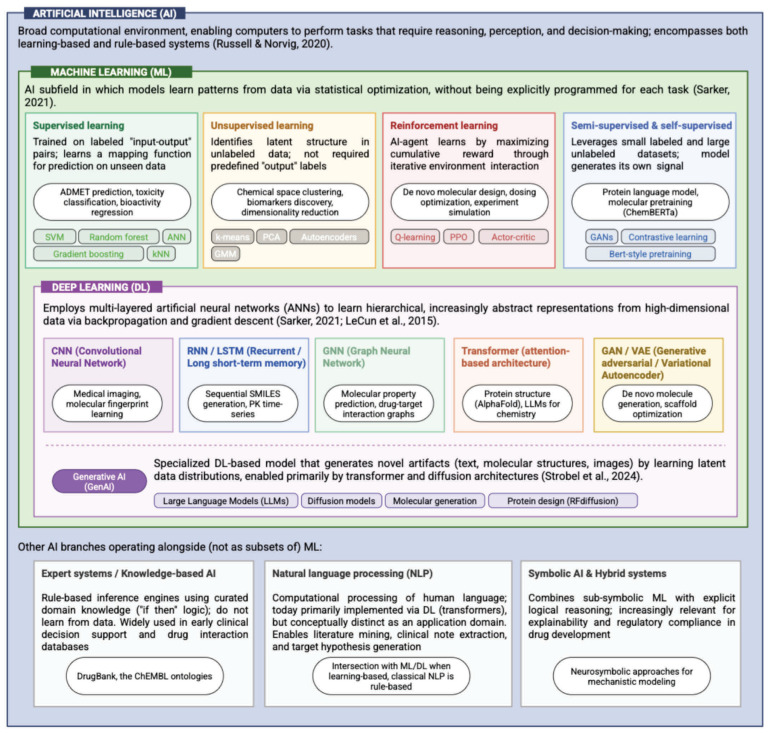
Hierarchical taxonomy of artificial intelligence and its principal methodological subtypes. *Abbreviations:* ADMET: absorption, distribution, metabolism, excretion, and toxicity; AI: artificial intelligence; ANN: artificial neural network; CNN: convolutional neural network; DL: deep learning; GAN: generative adversarial network; GenAI: generative artificial intelligence; GMM: Gaussian mixture model; GNN: graph neural network; k-NN: k-nearest neighbors; LLM: large language model; LSTM: long short-term memory; ML: machine learning; NLP: natural language processing; PCA: principal component analysis; PK: pharmacokinetics; PPO: proximal policy optimization; RNN: recurrent neural network; SMILES: simplified molecular input line entry system; SVM: support vector machine; VAE: variational autoencoder. *References for figure content:* [49,50,51,52,53,54,55,56].

In addition, beyond the foundational “paradigms” of ML and DL, artificial intelligence includes several specialized subtypes, each addressing distinct computational challenges with tailored algorithmic approaches (Figure 1).

For example, computer vision (CV) is dedicated to enabling computers to interpret and understand visual information from the real world [57]. Mathematically, CV tasks (e.g., object recognition [58], image segmentation [59], scene reconstruction [60], etc.) often involve complex transformations of pixel data, leveraging techniques from linear algebra, differential geometry, and statistical pattern recognition to extract meaningful features and assign semantic labels [61]. On the other hand, natural language processing (NLP) focuses on the interaction between computers and human language. It aims to enable machines to understand, interpret, and generate natural language [62]. NLP models frequently employ statistical methods, graph theory, and deep learning architectures (e.g., recurrent neural networks, transformers) to analyze linguistic structures, extract entities, and infer sentiment, often relying on probabilistic models to manage the inherent ambiguity of human communication [63]. Fuzzy logic (FL), in contrast to classical Boolean logic, operates on degrees of truth rather than absolute “TRUE/FALSE” values, allowing for the representation and processing of imprecise or uncertain information [64]. Its mathematical framework is built upon fuzzy set theory, where elements have degrees of membership to sets, typically represented by a membership function:

$$\mu_{A}\left(X\right)\in \left[0,1\right],$$

which assigns each element ***X*** a real-valued membership grade in the fuzzy set ***A*** [65].

This approach is particularly valuable in control systems and decision-making processes where inputs are inherently vague or subjective, providing a robust mechanism for handling real-world complexities that defy crisp logical definitions.

### 2.1. The Evolution of Artificial Intelligence in Drug Development

The historical evolution of integrating computational methods into the drug development process reveals a non-linear progression from descriptive analysis to predictive modeling, a shift that commenced long before the current “digitalization” and AI surge.

A transformation occurred in the 1960s with the advent of Quantitative structure–activity relationships (QSAR), pioneered by C. Hansch and T. Fujita, which for the first time mathematically linked chemical structure to biological response through descriptors of lipophilicity and electron density [66]. Concurrently, in the mid-1960s, the DENDRAL project at Stanford University laid the groundwork for expert systems. The core concept was the automation of the mass spectrometry data (with further mathematical interpretation) for organic compounds and the demonstration of the feasibility of encapsulating specific chemical knowledge into algorithmic forms [67]. These iterations, though constrained by the computational power of their era, established the conceptual foundation for rational drug design, which evolved in the 1980s and 1990s towards statistical machine learning, providing initial automation for target identification and virtual screening stages [68].

The 1990s marked a transformation from the theoretical basis of QSAR toward the practical approaches of the first “Big Data” wave in pharmacology (it was catalyzed by the industrialization of high-throughput screening (HTS) and combinatorial chemistry) [69,70]. This period demanded a move from sequential testing to parallel processing, where computational algorithms were tasked with navigating vast chemical libraries synthesized through techniques like split-and-pool synthesis. The emergence of Lipinski’s “Rule of Five” in 1997 represented a milestone in this period, providing a computational heuristic to filter “drug-like” molecules and signaling a broader shift towards integrating ADME (Absorption, Distribution, Metabolism, and Excretion) considerations at the earliest stages of lead identification [71,72].

The early 2000s were defined by the completion of the Human Genome Project [73], which expanded the landscape of potential therapeutic targets [74]. This scientific explosion necessitated the maturation of bioinformatics, bioengineering, and virtual screening, where molecular docking algorithms like AutoDock and GOLD began to simulate protein–ligand interactions with increasing fidelity [75,76,77]. Furthermore, the rise of computational biology systems and network pharmacology during this decade challenged the traditional “one drug, one target” dogma, employing graph theory to model the complex, interconnected pathways of human disease, thereby paving the way for more holistic target prioritization strategies [78].

By the 2010s, the field underwent a “Deep Learning Revolution”, triggered by the success of convolutional neural networks in image recognition, which were rapidly adapted for molecular modeling [79]. The Merck Molecular Activity Challenge in 2012 demonstrated that deep neural networks (DNNs) could consistently outperform traditional machine learning methods in predicting biological activity [80,81]. This decade also saw the rise of Generative AI, with variational autoencoders (VAEs) and generative adversarial networks (GANs) enabling the de novo design of molecules with optimized properties, effectively moving the field from “searching” the chemical space to “creating” within it [82].

The technological momentum culminated in the 2020s with the resolution of the protein folding problem by AlphaFold 2, an achievement recognized by the 2024 Nobel Prize in Chemistry awarded to Demis Hassabis and John Jumper, alongside David Baker for computational protein design [83,84]. Simultaneously, the 2024 Nobel Prize in Physics was awarded to John Hopfield and Geoffrey Hinton, acknowledging the fundamental neural network architectures that underpin these biological breakthroughs [85]. These accolades reflect a broader recognition of “digital biology” as a core pillar of modern science.

In this current landscape, the focus has shifted towards Agentic AI, exemplified by companies like BIOPTIC, founded by Andrey Doronichev [86]. Moving beyond static models, BIOPTIC utilizes multi-agent systems that autonomously plan, reason, and execute complex drug discovery workflows, effectively acting as “digital employees” that integrate scientific modeling with strategic due diligence. Their proprietary models—B1 for ligand-based screening, B2 for sequence-based binding prediction, and B3 for universal ADMET forecasting—aim to condense the traditional development cycle into a mere 3 years [87]. Altogether, this progression from 1960s expert systems to 2020s autonomous agents represents a fundamental re-engineering of the drug development pipeline, aiming for a more predictable, engineering-driven approach to human health.

### 2.2. The Global Jurisprudential Landscape of Artificial Intelligence in Healthcare and Drug Development

The systemic integration of AI into healthcare and drug development has necessitated a profound evolution in legal and regulatory frameworks worldwide. This transition reflects a global imperative to harness technological innovation while safeguarding public health, ensuring ethical deployment, and maintaining robust data governance. The chronological unfolding of these regulatory responses reveals a complex interplay of national priorities, technological readiness, and a growing consensus on the need for a unified, harmonized approach.

Early regulatory efforts, predating the widespread adoption of AI, primarily focused on the oversight of software as a medical device (SaMD) and the establishment of data privacy standards [88,89]. In the United States, for instance, the Health Insurance Portability and Accountability Act (HIPAA) of 1996 laid foundational rules for protecting patient data, inadvertently setting a precedent for future AI data governance [90,91]. As digital health solutions gained traction in the 2010s, regulatory bodies began to issue specific guidance. The US Food and Drug Administration (FDA), recognizing the unique characteristics of continuously learning algorithms, introduced its “Artificial Intelligence and Machine Learning (AI/ML) Software as a Medical Device (SaMD) Action Plan” in 2021, moving towards an adaptive regulatory framework capable of overseeing algorithms that evolve post-market [92]. This pragmatic approach was further solidified by the 2023 executive order on “Safe, Secure, and Trustworthy Development and Use of Artificial Intelligence”, which mandated federal agencies, including the Department of Health and Human Services (HHS), to establish safety programs for AI in clinical settings [93].

Parallel to these developments, the European Union embarked on a more comprehensive, “principles-based” approach. Initial discussions on AI ethics, culminating in the Ethics Guidelines for Trustworthy AI (2019) [94], paved the way for the landmark EU Artificial intelligence Act (2024) [95]. This pioneering legislation, the world’s first comprehensive AI law, adopts a stringent risk-based classification, categorizing most AI systems in healthcare and drug development as “high-risk” [96,97]. This designation imposes rigorous requirements for conformity assessments, data quality, transparency, and human oversight, profoundly influencing member states. For example, Germany’s Digital Healthcare Act (DVG, 2019) created a unique reimbursement pathway for digital health applications [98], while France’s Health Data Hub (2019) centralized medical data for AI research under strict GDPR compliance [99]. Other EU nations have similarly integrated AI into their national health strategies, emphasizing ethical AI and transparency in clinical decision support systems [100].

Across the globe, other major economies have forged distinct, yet often converging, regulatory paths. The United Kingdom has adopted a “pro-innovation approach to AI regulation”, outlined in its 2023 White Paper, favoring sector-specific oversight, with the Medicines and Healthcare products Regulatory Agency (MHRA) adapting its framework for AI-enabled medical devices [101].

In Asia, China has balanced aggressive AI development with a robust ethical framework, exemplified by its Ethical Norms for New Generation AI (2021) and the Administrative Measures for Generative AI Services (2023), which impose strict requirements on data veracity and medical content [102,103,104]. Japan, through its AI Strategy (2019) and the Pharmaceuticals and Medical Devices Agency (PMDA), has actively promoted the “Sakigake” designation to accelerate the approval of innovative AI-driven drugs and devices, emphasizing “human-centered” AI principles [105,106,107].

Other regions have also rapidly advanced their regulatory landscapes. Canada introduced the Artificial Intelligence and Data Act (AIDA) as part of Bill C-27 (2022), focusing on high-impact AI systems and aligning Health Canada’s guidelines with international standards for SaMD [108]. In Russia, the National Strategy for the Development of AI (2019) led to the implementation of Experimental Legal Regimes (ELR) in 2020, allowing for controlled testing of AI in healthcare [109]. By 2024, the government applied national standards for AI in clinical medicine [110]. Kazakhstan has emerged as a regional leader, enacting its first comprehensive AI Law (2025/2026) in Central Asia and integrating AI into its national “digital health roadmap” to streamline e-governance medical services [111].

In Latin America, Brazil’s AI Bill (Marco Legal do IA) is currently undergoing Senate review (2023–2024), aiming to balance innovation with civil rights in the health sector [112], while Mexico is developing preliminary bills for AI in public services and health [113].

On the African continent, South Africa released its National AI Policy Framework (2024), seeking to align AI deployment with the Protection of Personal Information Act (POPIA), addressing the unique socio-economic and ethical considerations pertinent to the region [114].

Across these diverse jurisdictions, a common thread is the recognition that AI in drug development requires specific regulatory attention. International bodies like the International Council for Harmonisation (ICH) and the World Health Organization (WHO) are increasingly advocating for global standardization of AI/ML in drug development and manufacturing [115,116]. This global jurisprudential evolution underscores a collective commitment to establishing robust, yet flexible, legal frameworks that can adapt to the rapid pace of AI innovation, ensuring that these transformative technologies are deployed safely, ethically, and effectively for the benefit of global health.

## 3. The Deep Learning Infrastructure of Modern Pharmaceutics

**Figure 2:**
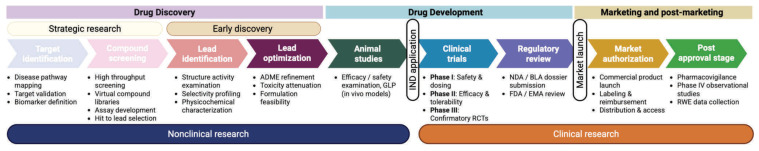
Overview of the drug development pipeline. *Abbreviations:* ADME: absorption, distribution, metabolism, and excretion; BLA: Biologic License Application; EMA: European Medicines Agency; FDA: US Food and Drug Administration; GLP: Good Laboratory Practice; IND: Investigational New Drug application; NDA: New Drug Application; RCT: randomized controlled trial; RWE: real-world evidence.

In the digital era of 2026, the general public is primarily acquainted with Artificial intelligence through the lens of conversational interfaces (e.g., ChatGPT, Claude, Grok, Gemini, etc.). These systems, while sophisticated in their ability to synthesize information or assist in administrative tasks, represent the “surface layer” of AI, optimized for natural language processing and token prediction [117,118]. In drug development, AI operates as a set of specialized methods of ML and DL that directly interface with core discovery stages (e.g., target identification, hit finding, lead optimization and others) [16]. Today, we believe that the integration of digital technologies can offer a “proactive” initiative that can influence the entire life cycle of drugs and all stages of drug development (Figure 2).

Nevertheless, despite ongoing improvements in discovery technologies and sustained growth in Research and Development (R&D) investment, the proportion of drug candidates that successfully progress to regulatory approval remains strikingly limited (approximately 10% of all candidate molecules; in simple terms, around one in ten) [119]. Most late-stage attrition is linked to issues rooted in the intrinsic physicochemical or biological characteristics of early compounds, which later manifest as inadequate efficacy or unanticipated safety liabilities [120].

Deep Learning and generative modeling now allow investigators to interrogate chemical spaces in the context of data volume. For example, the multibillion-molecule Enamine REAL Space helps identify candidates with improved ADME characteristics and more favorable interaction profiles [121]. This shift from exploratory search toward the deliberate computational design of molecular diversity reflects a broader digital transformation aimed at narrowing the long-standing divide between early discovery and clinical success.

The transition from exploratory R&D to formal preclinical development represents a critical point in pharmaceutical sciences. At this stage, researchers must distill millions of data points into a singular, viable drug candidate. This process is mediated by an advanced ecosystem of scientific databases and geometric modeling tools that provide the “ground truth” necessary for regulatory-grade evidence. Unlike general web-scale datasets, scientific databases are curated repositories that ground AI models in a practical way.

For example, DrugBank is a comprehensive resource that bridges the gap between chemical structures and clinical information. It contains detailed data on over 14000 drug entries, including FDA-approved small molecules and biotech drugs, providing the necessary pharmacokinetic (PK) and pharmacodynamic (PD) profiles required for preclinical modeling [122].

Another representative resource is PubChem, a database maintained by the U.S. National Institutes of Health that constitutes the largest openly accessible repository of chemical information worldwide. Containing more than 110 million distinct compounds, it functions as a central platform for high-throughput virtual screening and for conducting structure–activity relationship analyses [123,124].

BindingDB is a specialized database dedicated to experimentally determined binding affinities between drug-like small molecules and protein targets. It provides quantitative potency measurements (e.g., *Ki*, *Kd*, and IC50) that constitute essential training data for deep learning models aimed at predicting target engagement [125].

To convert the raw datasets into machine-learning-ready architectures, specialized tools have become indispensable. One of these is Graphein—a prominent Python library that enables the transformation of biological data from the Protein Data Bank (PDB) into geometric deep learning graphs. By representing proteins as graphs rather than static 3D coordinates, Graphein allows models to capture the topological and structural nuances of molecular interactions that are often lost in traditional modeling [126]. Furthermore, the integration of XBRLCA (eXtensible Business Reporting Language for Clinical Analysis) has begun to play a pivotal role in the “due diligence” phase of the R&D to preclinical transition. While traditionally used for financial transparency, its application in clinical data tagging ensures that preclinical results are standardized and interoperable across global regulatory frameworks [127]. This standardization is crucial for the quality control stages of AI platforms, allowing for the automated audit of numeric plots and trial data to mitigate the risk of clinical failure.

**Figure 3:**
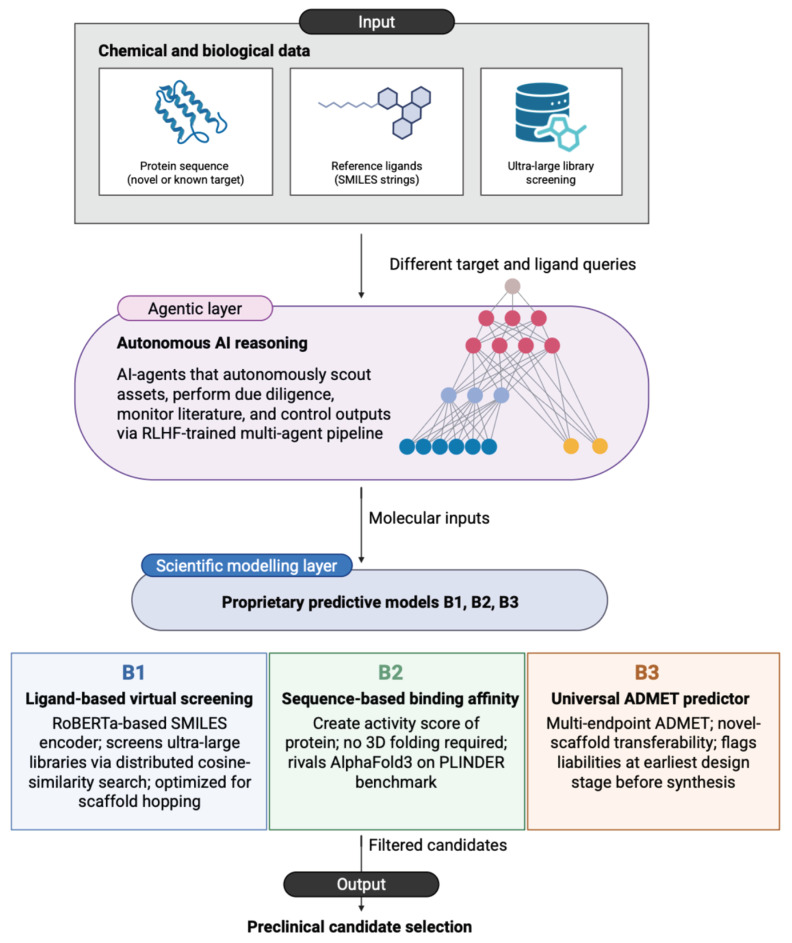
Computational workflow of the BIOPTIC AI platform for accelerated drug discovery. *Abbreviations:* ADMET: absorption, distribution, metabolism, excretion, and toxicity; AI: artificial intelligence; PLINDER: protein–ligand interactions dataset for benchmarking; RLHF: reinforcement learning from human feedback; SMILES: simplified molecular input line entry system.

A tangible example of deep integration of AI is seen in the achievements of BIOPTIC (Figure 3). We have previously outlined this company’s broader role in advancing the field; here, however, we refine that discussion by focusing on specific mechanistic details. Moving beyond the theoretical, BIOPTIC’s B1 model has successfully navigated the vast chemical space to identify novel LRRK2 inhibitors. These molecules are critical leads for Parkinson’s disease, targeting the G2019S mutation with dissociation constants (Kd) as low as 110 nM (a level of potency that significantly exceeds traditional screening outputs) [128]. In addition, beyond neurodegeneration, the platform’s “Agentic” reasoning layer has been deployed to scout and validate assets for complex oncological targets. By utilizing its B3 ADMET predictor, BIOPTIC has been able to flag toxicological liabilities at the earliest design stage, effectively condensing the R&D to preclinical timeline from years to months. Their discovery of novel, structurally diverse scaffolds for kinase inhibition marks a shift from incremental drug design to the automated discovery of first-in-class therapeutic agents. This transition is anchored by their PLUMBER benchmark, which utilizes 1.8 million binding affinity points to ensure that their “digital employees” operate with a level of scientific accuracy that rivals human scientists [87,129].

## 4. AI Integration Across the Pharmaceutical Product Lifecycle

**Figure 4:**
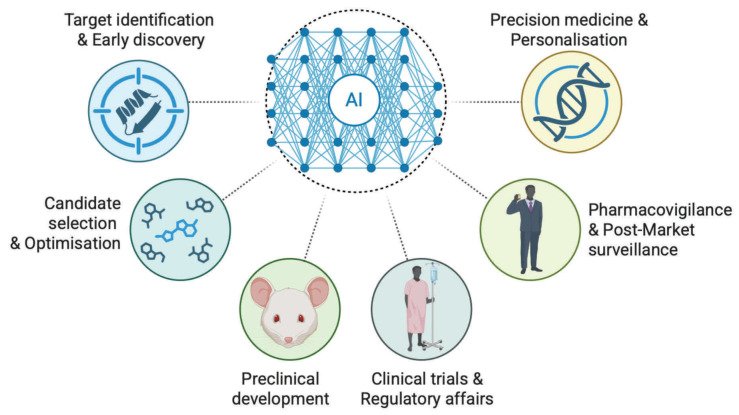
Applications of artificial intelligence across the pharmaceutical product lifecycle.

Drug development is a complex sequence of steps that requires substantial time and investment and carries a high probability of failure. The earliest phases of research continue to depend on laboratory experimentation, and many promising candidates do not progress further because critical shortcomings remain unnoticed at these initial stages [130]. Digital technologies can potentially be applied at every stage of the pharmaceutical product lifecycle (PPL) (and in some settings are already integrated in a “trial mode”): for example, supporting early discovery through data-driven hypothesis generation and target assessment [131]; strengthening development by refining candidate selection and modeling product quality [132,133]; and enhancing the later regulatory and clinical phases through structured evidence integration and continuous monitoring frameworks (Figure 4) [134,135]. It is important to recognize that computational methods cannot substitute for experimental validation completely, although they can reduce uncertainty in early exploration and help direct research toward the most promising hypotheses. Nevertheless, any broader integration of AI into early drug development should be approached cautiously, as predictive models remain limited by data quality, methodological constraints, and the essential role of empirical confirmation.

Whether AI could eventually assume responsibilities traditionally assigned to early discovery remains an open and actively discussed issue. Although current models demonstrate notable progress, their reliability is still bounded by the scope and quality of underlying data, as well as by the inherent uncertainties of biological systems. Experimental investigation therefore remains indispensable, and computational approaches are best viewed as strengthening early decision-making rather than replacing established scientific workflows (at least for today). From this standpoint, AI contributes by refining how information is generated, prioritized, and interpreted, creating a foundation for its systematic integration across the successive stages of the PPL.

### 4.1. AI in Drug Discovery

Drug discovery represents the foundational phase in the development of original drugs, encompassing a rigorous sequence of scientific inquiry that transitions from basic biological understanding to the identification of viable clinical candidates. This initial stage is traditionally structured into a series of interrelated milestones, including target identification, target validation, lead discovery, and lead optimization [136]. The primary objective of this phase is to isolate a pharmacologically active substance that demonstrates high affinity for a specific disease-associated target while maintaining a favorable safety profile. As the pharmaceutical industry faces increasing R&D costs and declining productivity, the drug discovery stage has become the primary focus for the integration of advanced computational methodologies aimed at reducing the high attrition rates associated with traditional “trial and error” experimentation [137].

**Figure 5:**
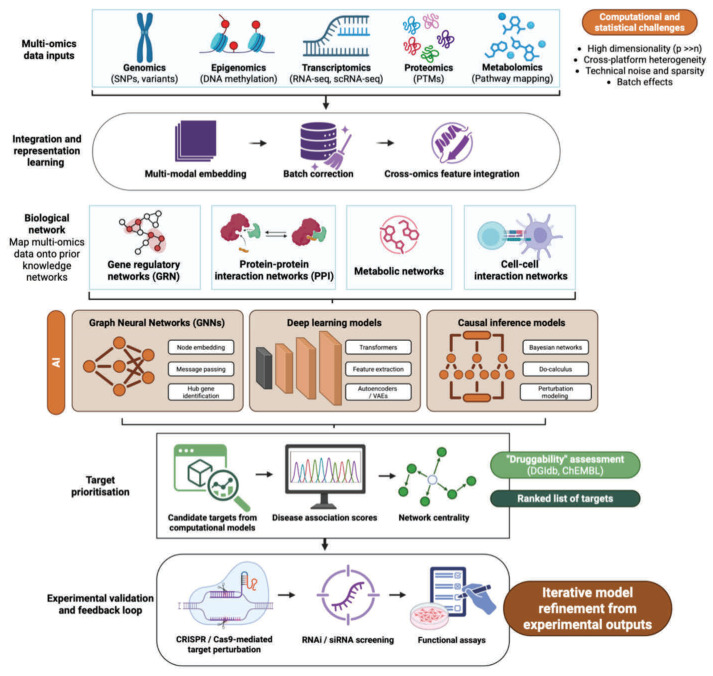
A multi-layered computational pipeline for network pharmacology-based target identification and experimental validation. The pipeline translates heterogeneous multi-omics data into experimentally validated therapeutic targets through six integrated computational layers: (1) Five molecular data modalities—genomic variants (SNPs), epigenomic DNA methylation, transcriptomic profiles (RNA-seq, scRNA-seq), proteomic post-translational modifications (PTMs), and metabolite pathway mapping—enter the pipeline simultaneously, each carrying a distinct biological signal obscured by high dimensionality (*p* >> n), cross-platform heterogeneity, technical noise, and batch effects; (2) Joint multi-modal embedding, batch correction, and cross-omics feature integration resolve these challenges into a unified molecular representation; (3) The representation is then projected onto four prior knowledge networks—GRN, PPI, metabolic, and cell–cell interaction networks—to recover biologically interpretable structure; (4) Three AI model classes extract complementary information from these networks: GNNs propagate molecular signals via message passing to identify hub genes; deep learning models (transformers, VAEs) compress network topology into latent representations; causal inference models (Bayesian networks, do-calculus) distinguish driver from passenger molecular events; (5) Candidate targets are ranked by disease association scores, network centrality, and “druggability” assessment (e.g., DGIdb, ChEMBL). (6) Top-ranked targets undergo experimental perturbation: CRISPR/Cas9-mediated knockout, RNAi/siRNA screening, and functional assays, with outcomes fed back to refine model parameters iteratively. *Abbreviations:* AI: artificial intelligence; ChEMBL: chemical database of bioactive molecules with drug-like properties; CRISPR/Cas9: clustered regularly interspaced short palindromic repeats/CRISPR-associated protein 9; DGIdb: Drug-Gene Interaction database; GNN: graph neural network; GRN: gene regulatory network; PPI: protein–protein interaction network; PTMs: post-translational modifications; RNAi: RNA interference; RNA-seq: RNA sequencing; scRNA-seq: single-cell RNA sequencing; siRNA: small interfering RNA; SNPs: single nucleotide polymorphisms; VAE: variational autoencoder.

The identification of disease-relevant targets has been fundamentally transformed by AI-driven multi-omics analysis and network pharmacology. Modern deep learning architectures, such as graph convolutional networks (GCNs), are now capable of sifting through vast genomic and proteomic datasets to pinpoint disease-associated genes with unprecedented precision (Figure 5) [138,139,140]. For instance, Zhang et al. (2025) demonstrated that AI-based target discovery platforms can rank disease-relevant genes by analyzing protein interaction networks, effectively reducing the time required for target validation compared to traditional biological assays [141]. These systems resolve cellular heterogeneity and map complex gene regulatory networks, identifying cell-type-specific targets that were previously inaccessible through bulk sequencing methods.

Predicting the binding affinity between a small molecule and its target protein is a critical bottleneck in lead discovery. AI models, particularly those utilizing 3D convolutional neural networks (3D-CNNs) and attention mechanisms, have shown superior performance in predicting drug–target interactions (DTIs) by learning directly from protein–ligand co-crystal structures [142,143,144].

Recent work by Liao et al. (2025) provides a systematic analysis of artificial intelligence methodologies for DTI and affinity (DTA) prediction, detailing how the transition from unimodal to multimodal frameworks improves the precision of binding assessments and accelerates the computational screening of therapeutic compounds [145]. Broader surveys of neural network-based approaches confirm this trend: CNNs, graph neural networks (GNNs), and Transformer-derived models consistently outperform traditional DTI methods, with hybrid architectures reaching AUROC values as high as 0.979 [146]. More advanced systems (e.g., Top-DTI), which integrate topological data analysis with large language model embeddings, further enhance predictive power across AUROC, AUPRC, sensitivity, and specificity metrics [147]. Methods like DTiGEMS+ report average AUPRC values around 0.92 on standard benchmarks, reflecting a substantial reduction in error relative to earlier computational frameworks [148]. Collectively, these advances allow researchers to triage millions of hypothetical protein–ligand pairs computationally, narrowing the experimental search space and reducing early development costs while maintaining dependencies on experimental validation.

On the other hand, the spatial arrangement and conformational flexibility of molecules dictate their biological activity and pharmacokinetic properties. AI-driven tools have revolutionized the prediction of protein structures, while generative models (e.g., VAEs) are used to explore the vast chemical space of small molecules. Hussein and Munirathinam (2026) noted that the integration of AI for optimizing molecular structures has enabled the identification of novel scaffolds that satisfy complex geometric constraints required for high-affinity binding [149]. These models can predict the absolute configuration of compounds with high accuracy, facilitating the design of stereochemically pure drug candidates.

When moving toward the biological interpretation of a candidate molecule, the integration of digital technologies likewise becomes an essential component of the analytical process. The optimization of ADMET parameters is essential for ensuring the clinical viability of a drug candidate [150,151].

Deep learning has reshaped this area by enabling models to learn molecular features directly from raw chemical structures rather than relying on predefined points. Graph-based neural architectures, in particular, represent molecules as node–edge graphs and learn spatial and electronic relationships that traditional fingerprints fail to capture [152]. This capability allows algorithms to uncover nuanced structure–property relationships and generalize predictions across broader regions of chemical space, including scaffolds not represented in the training set [153]. The transition from handcrafted features to data-driven molecular representations has addressed long-standing limitations in cheminformatics and has opened new directions for virtual screening and early risk assessment [154]. Recent platforms (e.g., Deep-PK) exemplify these advances by leveraging graph neural networks and graph-derived signatures to predict dozens of pharmacokinetic and toxicity endpoints simultaneously, demonstrating improved accuracy across 73 measurable properties and supporting model-guided molecular refinement [155]. Together, these developments indicate that ADMET modeling, once a bottleneck in early discovery, is increasingly supported by computational systems capable of providing reliable and interpretable predictions prior to experimental testing.

### 4.2. De Novo Molecular Design

One of the increasingly significant directions within the PPL is the use of computational algorithms for de novo molecular design. This area has undergone profound evolution over the past three decades. For example, Mouchlis and colleagues (2021) describe de novo design as a framework in which molecules are assembled from atomic or fragment-level components without requiring predefined structural analogs [156]. Meyers et al. (2021), in turn, emphasize that this field has been reshaped by the emergence of deep learning, particularly through atom-based, fragment-based, and reaction-based generative approaches [157].

A growing body of work illustrates the practical utility of these models. Tiago Sousa et al. (2021) showed that recurrent neural networks, autoencoders, and generative adversarial networks can be trained on curated datasets and subsequently refined through transfer learning, Bayesian optimization, or reinforcement learning to produce compounds with specific pharmacological profiles [158]. Practical tools (e.g., AlvaBuilder) demonstrate that these approaches are no longer restricted to theoretical studies. Mauri and Bertola (2023) describe how end users can design candidate molecules directly from descriptor-based models and QSAR/QSPR predictions [159].

Several recent studies highlight the scientific breadth of AI-driven de novo design. For example, Srinivasan and coauthors (2021) reported an integrated workflow combining Monte Carlo tree search with neural networks to propose inhibitors of the SARS-CoV-2 spike protein, generating hundreds of candidates that exceeded the performance of reference FDA-approved compounds [160]. In addition, Blaschke et al. (2020) addressed a well-known “bottleneck” in reinforcement learning by introducing a memory-assisted variant that expanded structural novelty in ligands for dopamine and serotonin receptors [161]. Complementing these advances, Lin et al. (2020) provided a systematic overview of generative adversarial networks and their applications in molecular design, dimensionality reduction, and peptide/protein engineering [162]. Atz et al. (2024), on the other hand, demonstrated that interactome-guided deep learning can produce ligands with nanomolar affinity for challenging targets such as GPCRs, effectively reversing the classical QSAR workflow by mapping desired biological activity back to chemical structure [163]. Although their work operates in a highly controlled computational space, it provides a compelling illustration of how AI-driven methods can expand the accessible chemical universe beyond empirically synthesized scaffolds.

Taken together, these findings show that de novo molecular design has moved from a supporting computational method to a core component of early drug discovery. By generating chemically diverse and target-focused candidates, it strengthens experimental workflows and helps streamline the initial stages of development.

### 4.3. Lead Optimization

Lead optimization is a critical stage of drug discovery in which scientists refine an initial active compound to achieve an acceptable balance of potency, selectivity, safety, pharmacokinetic properties, and other parameters [164,165]. This step aims to convert an early “hit” into a molecule that has a realistic chance of progressing toward clinical evaluation. Artificial intelligence increasingly assists this process by evaluating large numbers of possible structural modifications and proposing changes that can enhance the overall profile of a lead compound [166].

An interesting 2025 study by Koirala M et al. demonstrates that deep-learning architectures are transforming lead discovery by enabling the autonomous generation of novel molecular scaffolds and the precise prediction of ADMET properties. By utilizing generative models for inverse QSAR, this approach allows for the targeted design of candidates with optimized bioactivity and physicochemical criteria, effectively mitigating the high costs and prolonged timelines of traditional drug development [167].

In simpler terms, lead optimization can be viewed as a systematic search for the best possible version of a promising molecule, where each modification is evaluated not in isolation but in terms of its contribution to the overall therapeutic potential.

### 4.4. Integrated Case Studies from Industry Leaders

The rapid adoption of AI across the pharmaceutical sector illustrates a digital transformation in how companies generate hypotheses, design molecules, evaluate clinical evidence, and manage large-scale R&D portfolios [168]. Although the specific implementations differ across companies (an important factor here is the legal environment in each specific country), a unifying theme emerges: AI is shifting from a supportive analytical tool to a structural component of modern drug development. The following case studies demonstrate how leading pharmaceutical companies are operationalizing this transition and highlight the strategic patterns that underpin an AI-enabled R&D ecosystem in BigPharma.

Pfizer provides one of the clearest examples of accelerated development through AI integration. The company used AI-driven discovery platforms during the development of Paxlovid^®^, enabling a markedly shorter research timeline and achieving a clinical success rate above the industry average [169]. Pfizer’s 2025 strategy places high-quality data at the center of all AI initiatives. It underscores the principle that the effectiveness of AI models depends directly on the relevance and integrity of the underlying datasets. By embedding AI throughout the R&D value chain, Pfizer aims to minimize development friction and shorten the path from concept to patient [170,171,172].

Merck & Co., Inc. (USA) has taken a complementary approach by deploying large language model-based “AI agents” to support scientific reasoning and early hypothesis refinement. In its 2026 collaboration with the Mayo Clinic, Merck emphasized the role of generative AI in reducing the time required to design and operationalize clinical studies. Internally, AI platforms automate routine analytic tasks, allowing research teams to focus on complex problems that require human insight and domain expertise. This model reflects a shift toward hybrid systems in which AI augments, rather than replaces, scientific decision-making [173].

Merck KGaA (Germany) launched AIDDison, an AI-enabled platform that provides API-based access to computational chemistry and molecular design tools, positioning AI as an embedded component of the R&D workflow [174].

Sanofi has adopted an “AI-first” organizational strategy that scales AI capabilities across all therapeutic areas. Through the tool “plai”, the company integrates AI into portfolio management, target identification, and molecule design. Partnerships with technology firm Owkin demonstrate Sanofi’s commitment to precision medicine, particularly in oncology and mRNA therapeutics, where multidimensional datasets and real-world evidence are essential for understanding patient heterogeneity [175,176,177,178].

AstraZeneca focuses on embedding AI into its digital health and evidence-generation infrastructure. By applying AI to characterize protein interaction networks and disease-associated genes, especially in oncology and cardiometabolic disorders, the company strengthens decision consistency and aligns clinical development with real-world therapeutic performance [179,180,181]. This integration also supports regulatory transparency, an increasingly important requirement as digital tools enter formal evaluation pathways.

Bayer’s digital transformation strategy highlights AI-driven methods (e.g., optimizing molecular structures, accelerating hit-to-lead and lead optimization processes). By leveraging large-scale biological and chemical datasets, Bayer advances candidates for complex conditions like chronic kidney disease and advanced cancers [182,183].

Together, these examples show that AI is reshaping the BigPharma research environment. Although each company pursues distinct priorities, they converge on the principle that AI “must be integrated, not added superficially” into the structure of R&D if it is to deliver meaningful scientific and clinical impact.

### 4.5. AI in Preclinical Trials

The preclinical stage of drug development establishes the first evidence base for safety and biological activity before human exposure [184]. Regulatory frameworks, depending on the legal environment of a particular country, require a structured set of studies: primary pharmacology, general and reproductive toxicity, pharmacokinetic and toxicokinetic evaluation, genotoxicity, and, when justified, assessments of carcinogenicity, immunotoxicity, phototoxicity, or dependence liability [185]. Although these datasets are limited in scope, they must be sufficient to anticipate adverse reactions that may occur during early clinical testing and to justify the design of “first-in-human” studies [186].

A persistent challenge is the translational gap: findings in vitro and in vivo often fail to predict human responses, largely because healthy, genetically uniform animals do not reflect human physiological heterogeneity [187,188,189]. Olson and colleagues (2000), in a retrospective analysis of 150 compounds across 12 pharmaceutical companies, found that rodent and non-rodent studies together predicted only 71% of human toxicities; for certain endpoints (central nervous system effects, gastrointestinal toxicity), concordance dropped below 50% [190]. Liu and Fan (2026) harmonized preclinical and clinical safety records for 7565 drugs across five species (mouse, rat, rabbit, dog, monkey) and identified 850 adverse events that reached statistical significance in both animals and humans. More interesting is that of the 26 system organ classes evaluated, only one—infections and infestations—exceeded a positive likelihood ratio of 10 for both small molecules and biologics. Nervous system disorders, psychiatric endpoints, and general systemic conditions fell below three. More concerning was the negative predictivity: when an adverse event was absent in animals, this provided almost no information about whether it would appear in humans. The inverse negative likelihood ratios clustered near one across nearly every endpoint and every species. Species choice shaped predictivity as much as algorithm choice. Non-human primates produced the highest median positive likelihood ratios (4.18 for small molecules; 3.74 for biologics). Dogs proved particularly informative for cardiovascular and gastrointestinal endpoints in exposure-controlled analyses; rabbits were informative for ocular and reproductive toxicities. Rodents contributed large volumes of training data but weaker translational signal for most organ systems. This pattern carries a direct implication for AI, because a model trained predominantly on rat hepatotoxicity data will learn to predict rat hepatotoxicity [191].

Whether that prediction transfers to human organs depends on biological factors that the algorithm itself cannot verify. The question, then, is not only which algorithm we choose, but what data we “feed” it and whether the volume of available preclinical records compensates for their limited human relevance, or merely scales the same translational uncertainty to a larger dataset.

At the same time, given the trend in preclinical research toward reducing the use of animals in efficacy and safety studies [192,193], there is particular interest in developing digital systems that can be used as alternatives to animal models. The major pharmaceutical companies (Bayer, Eli Lilly, Genentech, Gilead, GSK, Merck Healthcare, and Roche) are collectively funding an attempt to build a virtual system similar in its biological nature to a dog. The NC3Rs virtual second species challenge, launched with £1.6 million awarded to Dr. Stephan Schaller’s team at esqLABS, asks a direct question: Can a computational model of canine organ physiology replace live dogs in chronic toxicity studies for new chemical entities? Currently, the proposed model is capable of simulating only one criterion: hepatic steatosis (which is undoubtedly a limiting factor). Although the search for and development of such models represent a cutting-edge field of science, real-world studies in dogs, on the other hand, generate data on dozens of organs, hundreds of clinical pathology parameters, electrocardiograms, ophthalmoscopic examinations, urinalysis panels, and daily observations of each animal’s behavior at every dose level [194,195]. This is precisely why it is extremely important to continue research in this direction.

Regulators therefore emphasize early identification of human-relevant risk indicators, including pharmacodynamic and metabolic characteristics, receptor-binding patterns, and structural similarity to substances known to produce dependence. These principles guide the need for additional studies and help determine whether extended toxicology or dependence-related assessments are required [196,197]. Critically, the “black box” nature of deep learning models in toxicology creates a validation gap; without mechanistic interpretability, AI-driven safety predictions often fail to satisfy the evidentiary standards required for regulatory-grade decision-making, particularly when predicting rare but catastrophic idiosyncratic reactions [198].

Artificial intelligence offers a means to strengthen the predictive value of preclinical evidence while remaining aligned with established toxicological requirements. AI-enabled new approach methodologies (NAMs) integrate chemical, biological, and toxicological data to improve human relevance without eliminating the need for animal studies. For example, stem-cell-derived platforms combined with ML models allow researchers to evaluate drug responses across diverse genetic backgrounds before in vivo testing [199,200,201].

Lee et al. (2025) showed that graph neural networks enhance the prediction of organ-specific toxicities, outperforming classical descriptor-based methods [202]. Lu et al. (2025) demonstrated that the ToxACoL framework improves cross-species toxicity extrapolation and reduces the need for extensive animal testing [203]. Experimental NAMs show similar progress: David Paik et al. (2020) used iPSC-derived endothelial models to detect cardiovascular effects relevant to specific patient groups [204], while organ-on-chip systems (e.g., “liver chip”) identified hepatotoxic compounds missed by traditional assays [205,206]. Reinforcement-learning approaches have also optimized dose-range selection [207,208]. In parallel, generative AI now embeds ADMET constraints into molecular design from the outset [209].

It is important to highlight that archived toxicogenomic databases have blind spots. They contain “dose/response” profiles only for compounds that someone, at some point, had put into a living animal. For example, Tox-GAN operates as a generative adversarial network trained on rat liver gene expression data from approximately 150 compounds in the Open TG-GATEs database [210]. The model produces outputs only within the chemical space its training set defines. Organs on chip (OoC) systems remove that constraint by generating original dose–response data from human tissue exposed to compounds with no prior experimental record.

**Figure 6:**
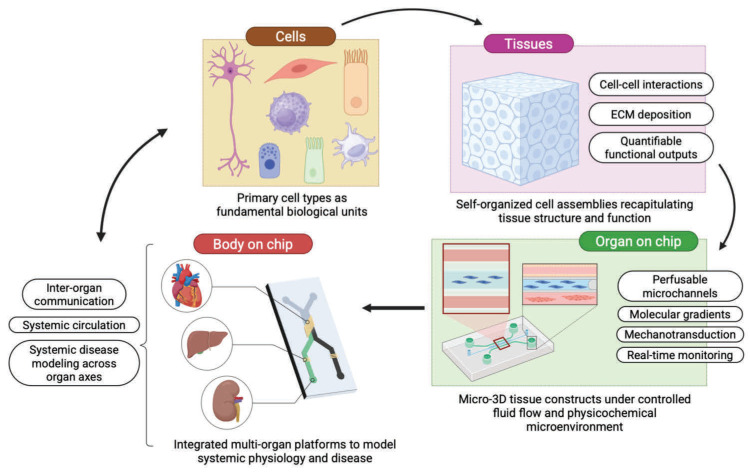
Hierarchical organization of biological and microengineering systems. This figure illustrates how four hierarchical levels replicate human physiology in vitro. Primary cell types self-organize into tissue structures characterized by intercellular interactions, extracellular matrix deposition, and measurable functional parameters. Using advanced microengineering technologies, tissues have been integrated into “organ on chip” devices under conditions of controlled fluid flow and a controlled physicochemical microenvironment. Organ modules can be combined into more complex systems, referred to in the literature as “organism on chip.” These systems already feature interorgan communication, systemic circulation, and disease modeling across all organs. *Abbreviations*: ECM, extracellular matrix.

An OoC is a polymer scaffold threaded with microfluidic channels that maintain living human cells under continuous fluid flow, replicating the mechanical forces and nutrient gradients that define organ function in vivo (Figure 6). Dai and colleagues (2023) showed that convolutional neural networks applied to “liver on chip” time-lapse microscopy classified hepatocyte injury phenotypes at spatial resolution exceeding manual histopathological scoring [211]. Another example: Bai and colleagues (2023) reported that integrated sensors within OoC platforms provide continuous data on metabolic activity. AI models process these signals to identify early toxicity markers, effectively creating a “digital twin” of the organoid that can predict long-term outcomes from short-term data [212]. At the multi-organ level, Erika Ferrari and Marco Rasponi (2021) connected liver and heart compartments in a pumpless gravity-driven platform and demonstrated that cyclophosphamide (a drug without direct cardiac activity) generates the cardiotoxic metabolite acrolein following hepatic metabolism, which reduced cardiomyocyte conduction velocity and beat frequency, and prolonged the QT interval—a cross-organ interaction that no single-tissue assay captures [213].

Overall, these developments indicate a shift toward a more predictive and human-centered preclinical environment.

### 4.6. AI in Clinical Trials

The clinical development phase constitutes the most resource-intensive and high-stakes segment of the pharmaceutical lifecycle, aimed at establishing the safety and therapeutic efficacy of a candidate molecule in human subjects. Traditionally, this process is structured into three sequential phases, governed by the rigorous standards of the International Council for Harmonisation (ICH) E6 Good Clinical Practice (GCP) guidelines. However, the latest revision, ICH E6(R3), marks a shift in approach by explicitly encouraging the integration of innovative trial designs and digital technologies to enhance efficiency and data reliability [214,215]. While the traditional linear progression remains a baseline, modern clinical research increasingly adopts “adaptive” and “seamless” designs, where phases may overlap or be modified based on interim data analysis, thereby accelerating the path to regulatory submission. Central to this process is the Helsinki Declaration, which mandates that all clinical research be grounded in ethical principles that prioritize the rights, safety, and well-being of participants over the interests of science or society [216,217,218,219]. Furthermore, any trial must be preceded by a rigorous prospective assessment of the benefit–risk ratio, ensuring that the anticipated societal and individual benefits justify the inherent risks and inconveniences to the subjects.

The emergence of software as a medical device (SaMD), particularly those incorporating AI and ML, has introduced new requirements for clinical evidence generation. For AI-based medical software, clinical data must be derived from patient populations and verified by accredited medical organizations through diagnostic, therapeutic, rehabilitative, or other workflows [220,221]. Regulatory bodies require a comprehensive analysis of both favorable and unfavorable clinical data, sourced from scientific literature, clinical experience, and dedicated trials, to form a robust conclusion on the device’s safety and effectiveness. This analysis must account for the device’s specific intended use, its potential risk class, and the unique characteristics of the target patient population [222,223].

The practical application of AI in clinical trials has demonstrated transformative potential across several domains. AI-driven screening of electronic health records (EHRs) has been shown to reduce recruitment timelines [224]. For instance, Yang Y. et al. (2022) utilized AI to identify eligible participants for Parkinson’s disease trials by detecting subtle digital biomarkers in gait and speech, significantly improving the precision of cohort selection [225]. However, the use of AI for cohort selection faces the “bottleneck” of data sparsity and population bias, which is important to keep in mind; models trained on historical EHR data often fail to generalize to underrepresented demographics, potentially compromising the equity and external validity of the trial results.

On the other hand, the use of AI to model control groups from historical clinical data and real-world evidence is gaining regulatory traction. IQVIA reported that synthetic control arms (SCAs) can reduce the number of patients required for the placebo arm in rare disease trials, thereby addressing ethical concerns regarding placebo administration in life-threatening conditions [226]. Nevertheless, the primary limitation lies in “unmeasured confounding”, as AI models cannot account for variables not captured in historical datasets, which may lead to biased estimates of treatment effects compared to traditional randomized arms.

Digital twins represent a complementary approach. The PIONEER platform by Evaxion Biotech utilizes generative AI and digital twin technology to personalize translational research. By simulating patient-specific responses to immunotherapies, the platform has enhanced the predictability of clinical outcomes in heterogeneous cancer populations [227,228,229].

AI algorithms are now employed for real-time monitoring of trial data to detect safety signals or data anomalies. A 2025 scoping review highlighted “silent trials”, where AI models are tested prospectively in non-interventional settings to validate their predictive accuracy before being integrated into active clinical decision-making [230].

The integration of wearable sensors and AI-based remote monitoring has facilitated the rise of decentralized clinical trials. ICH E6(R3) specifically addresses these decentralized elements, for example, remote consent and monitoring. These innovations have been shown to increase participant diversity and reduce dropout rates by making trials more accessible to broader geographic and demographic groups [215].

In conclusion, the integration of AI into clinical trials represents more than a technological upgrade; it is a fundamental shift toward a more efficient and patient-centric model of evidence generation. By leveraging high-dimensional data and predictive modeling, researchers can now navigate the complexities of human biology with greater precision, ensuring that only the most promising and safe therapies reach the market. However, the long-term success of this shift depends on overcoming the systemic challenges of data quality, model interpretability, and the establishment of harmonized global regulatory standards that can keep pace with the rapid evolution of agentic AI systems.

### 4.7. AI in Pharmaceutical Marketing

Marketing activity within the pharmaceutical sector serves as a critical entrepreneurial function, ensuring the sustainable and competitive development of market entities by aligning internal capabilities with the dynamic external environment. According to the American Marketing Association (AMA), marketing research is defined as the systematic search, collection, processing, and integration of information related to the marketing of goods and services [231]. In the contemporary pharmaceutical landscape, this systematic activity is increasingly driven by artificial intelligence, which transforms market research from a retrospective analysis into a predictive and proactive strategic tool. Market research, in this context, is viewed as a systematic endeavor aimed primarily at analyzing the market’s capacity to reproduce the turnover of pharmaceutical products, encompassing both prescription (Rx) and over-the-counter (OTC) segments [232,233].

The integration of AI into marketing research allows for a more granular understanding of market dynamics, including segmentation, price fluctuations, and demand patterns [234,235]. By leveraging ML algorithms, pharmaceutical companies can now conduct high-fidelity research into specific drug segments, analyzing distribution by active substances, identifying therapeutic analogs, and modeling the impact of potential substitutes [236]. This technological step is not an optimization of existing processes; it represents a fundamental redefinition of how pharmaceutical entities interact with their stakeholders. The global AI in pharmaceutical market, valued at approximately $1.94 billion in 2025, is projected to expand to $16.49 billion by 2034, reflecting a robust compound annual growth rate (CAGR) of 27%. This growth underscores the transition toward an “intelligent” marketing ecosystem where data-driven insights form the basis for all strategic and tactical decisions [237,238].

Marketing research operates as the foundation for developing comprehensive strategies and programs aimed at enhancing organizational efficiency. The ability of AI to process vast datasets enables BigPharma companies to anticipate market shifts and allocate resources with unprecedented precision. For instance, empirical studies have demonstrated that AI-enhanced Enterprise resource planning (ERP) and Customer relationship management (CRM) systems significantly improve forecasting accuracy and inventory management, leading to better alignment with fluctuating market demands [239,240]. Consequently, the modern pharmaceutical marketing function is characterized by a shift from “push-based” promotional models to “pull-based” engagement strategies, where the focus is on providing value and relevance to both healthcare professionals (HCPs) and patients.

## 5. Evolving Frontiers and Unresolved Challenges

The transformative potential of digital technologies (in particular, the integration of AI) in drug development, while undeniable, is currently navigating a complex landscape fraught with systemic challenges that impede its full, synergistic integration. However, the challenges are not only technical; they are deeply embedded within different steps (e.g., regulatory frameworks, data ecosystems, and organizational structures), demanding a nuanced and often “out-of-the-box” approach to resolution. The promise of AI to accelerate drug discovery and optimize clinical trials often collides with the fragmented reality of global pharmaceutical regulation and the inherent complexities of data governance [241].

One of the most salient challenges is the regulatory divergence across international jurisdictions. Drug development is inherently a cross-functional and cooperative endeavor, yet the regulatory bodies overseeing AI applications (e.g., FDA, EMA (European Medicines Agency)) have adopted distinct philosophical approaches to oversight [115]. The FDA, for instance, has historically favored a flexible, case-specific model, evolving its guidance through direct engagement with industry applications. This “artisanal regulation” allows for adaptability but can introduce uncertainty regarding general expectations, particularly for novel AI-driven solutions [242,243,244,245]. In contrast, the EMA employs a more structured, risk-tiered approach, establishing explicit requirements across different development phases. While this provides clearer pathways to market, it may also extend innovation cycles and create compliance burdens, especially for smaller entities [115]. How can an industry that thrives on global collaboration effectively navigate such disparate regulatory philosophies without stifling innovation or compromising patient safety? (The question remains whether a truly harmonized global framework is an aspirational ideal or a pragmatic necessity in an increasingly AI-driven world).

This regulatory fragmentation is further compounded by the intricate issues surrounding data quality and data security. Can the industry truly unlock the full potential of AI without a concerted, global effort to standardize data infrastructure and ensure ethical data governance? The answer likely lies in collaborative initiatives that transcend national borders and institutional silos.

The deployment of AI in drug development raises fundamental questions about the integrity and interoperability of the vast datasets required for model training and validation [246]. AI systems, particularly those operating as “black boxes”, introduce unprecedented complexity and opacity, making transparent validation and oversight challenging [247,248]. The risk of AI models amplifying errors or pre-existing biases in their training data is a significant concern, raising questions about the generalizability of insights across diverse patient populations and the ethical implications of biased outcomes [249,250].

Moreover, the volume and heterogeneity of data (e.g., genomic sequences, RWE, EHRs) present formidable data integration challenges. Many pharmaceutical companies struggle with the lack of standardized “AI-ready” data, which hinders the seamless flow of information necessary for robust AI model development and deployment [251,252]. The US healthcare system, for example, faces significant barriers to AI adoption due to regulatory fragmentation between HIPAA and FDA frameworks, particularly concerning data de-identification and consent for secondary use, which leads to operational, legal, and economic burdens [253,254].

On the other hand, the vision of a fully AI-integrated drug development pipeline, while compelling, is often hindered by the inherent difficulties in fostering cross-functional cooperation and aligning diverse stakeholder interests. Drug development, by its very nature, is a collaborative endeavor involving chemists, biologists, clinicians, regulatory experts, and now, data scientists and AI engineers. The effective integration of AI necessitates not only the development of robust AI models but also the creation of seamless interfaces and communication protocols that bridge these traditionally disparate disciplines [255]. Is the pharmaceutical industry sufficiently agile to embrace such restructuring, or will legacy systems and entrenched practices continue to impede true systemic integration?

Furthermore, the discussion surrounding AI integration frequently converges on the critical issue of workforce transformation and the potential for job displacement. Concerns about AI rendering human roles obsolete are prevalent, echoing historical anxieties during periods of significant technological upheaval. However, a more pragmatic perspective suggests that the pharmaceutical industry is poised for a transformation analogous to the manufacturing transformation witnessed during the industrial revolution [1,256,257]. While certain tasks may be automated, the overall impact is likely to be a redefinition of roles, demanding new skill sets and fostering a more symbiotic relationship between human expertise and AI capabilities. For instance, AI will increasingly augment human decision-making, allowing researchers to focus on higher-level cognitive tasks, critical thinking, and creative problem-solving, rather than repetitive data analysis [258]. This shift necessitates proactive investment in upskilling and reskilling initiatives to prepare the existing workforce for these evolving demands.

In conclusion, the journey towards a fully AI-integrated drug development environment is not a linear progression but a complex, multidimensional evolution. The challenges that exist today are not insurmountable barriers but rather catalysts for a more robust and resilient pharmaceutical ecosystem. The industry is at an inflection point, where the narrative is shifting from viewing AI as a disruptive force to embracing it as a synergistic partner. The future of drug development will likely be defined not only by a binary choice between human intellect and artificial intelligence but also by their seamless integration. As the industry continues to navigate this intricate landscape, the most successful organizations will be those that foster a culture of continuous learning, champion cross-functional collaboration, and invest in the human capital that will ultimately guide the ethical and effective deployment of AI. The path forward demands a concerted effort from all stakeholders (e.g., companies, regulators, industry leaders, researchers) to build a human-centric future, ensuring that the transformative power of AI is harnessed to its fullest potential for the benefit of patients worldwide.

## Data Availability

No new data were created or analyzed in this study.

## Abbreviations

The following abbreviations are used in this manuscript:

3D-CNN	3D convolutional neural network
ADMET	Absorption, Distribution, Metabolism, and Excretion
AI	Artificial intelligence
AIDA	The Artificial Intelligence and Data Act
AMA	American Marketing Association
ANN	Artificial neural network
CV	Computer vision
DL	Deep learning
DTA	Drug–target affinity
DTI	Drug–target interaction
DNN	Deep neural network
EMA	European Medicines Agency
ERP	Enterprise resource planning
FDA	The US Food and Drug Administration
FL	Fuzzy logic
GAN	Generative adversarial network
GCN	Graph convolutional network
GCP	Good Clinical Practice
GNN	Graph neural network
CRM	Customer relationship management
HCP	Healthcare professional
HHS	Health and Human Services
HIPAA	The Health Insurance Portability and Accountability Act
HTS	High-throughput screening
ICH	The International Council for Harmonisation
MHRA	The Medicines and Healthcare products Regulatory Agency
ML	Machine learning
NAM	New approach methodology
NLP	Natural language processing
OoC	Organs on chip
PD	Pharmacodynamic
PK	Pharmacokinetic
PMDA	The Pharmaceuticals and Medical Devices Agency
PPL	Pharmaceutical product lifecycle
QSAR	Quantitative structure–activity relationships
R&D	Research and Development
ReLU	Rectified linear unit
SaMD	Software as a medical device
VAE	Variational autoencoder
WHO	The World Health Organization
XBRLCA	eXtensible Business Reporting Language for Clinical Analysis

## References

[1] Paul D., Sanap G., Shenoy S., Kalyane D., Kalia K., Tekade R.K. (2021). Artificial intelligence in drug discovery and development. Drug Discov. Today.

[2] Mitchell J.B. (2018). Artificial intelligence in pharmaceutical research and development. Future Med. Chem..

[3] Agrawal S. (2026). AI in Healthcare: Current Investment Trends. Qubit Capital. https://qubit.capital/blog/ai-healthcare-investment-trends.

[4] Bessemer Venture Partners (2026). State of Health AI 2026. https://www.bvp.com/atlas/state-of-health-ai-2026.

[5] (2025). Fortune Business Insights. Artificial Intelligence in Healthcare Market Report. https://www.fortunebusinessinsights.com/industry-reports/artificial-intelligence-in-healthcare-market-100534.

[6] Oualikene-Gonin W., Jaulent M.C., Thierry J.P., Oliveira-Martins S., Belgodère L., Maison P., Ankri J., Scientific Advisory Board of ANSM (2024). Artificial intelligence integration in the drug lifecycle and in regulatory science: Policy implications, challenges and opportunities. Front Pharmacol..

[7] Paerhati Y., Aikebaier A., Dilimulati D., Baishan A., Yusufujiang N., Qiu X., Wusiman Y., Zhou W. (2026). Rethinking Nature’s Pharmacy: AI Era and Natural Product Drug Discovery. Pharmaceutical.

[8] Bate A., Michael Tregunno P. (2026). How is AI developing in pharmacovigilance?. Ther. Adv. Drug Saf..

[9] Mennella C., Maniscalco U., De Pietro G., Esposito M. (2024). Ethical and regulatory challenges of AI technologies in healthcare: A narrative review. Heliyon.

[10] Sinha S., Lee Y.M. (2024). Challenges with developing and deploying AI models and applications in industrial systems. Discov. Artif. Intell..

[11] Ciallella H.L., Zhu H. (2019). Advancing Computational Toxicology in the Big Data Era by Artificial Intelligence: Data-Driven and Mechanism-Driven Modeling for Chemical Toxicity. Chem. Res. Toxicol..

[12] Abbas M.K.G., Rassam A., Karamshahi F., Abunora R., Abouseada M. (2024). The Role of AI in Drug Discovery. Chembiochem.

[13] Pavlović E., Dutta S., Garssen J., Folkerts G. (2026). Beyond the cage: The rise and promise of non-animal models in biomedical research. Eur. J. Pharmacol..

[14] Ajisafe O.M., Adekunle Y.A., Egbon E., Ogbonna C.E., Olawade D.B. (2025). The role of machine learning in predictive toxicology: A review of current trends and future perspectives. Life Sci..

[15] Badani A., de Moraes F.Y., Vollmuth P., Chung C., Mansouri A. (2025). AI and innovation in clinical trials. npj Digit. Med..

[16] Serrano D.R., Luciano F.C., Anaya B.J., Ongoren B., Kara A., Molina G., Ramirez B.I., Sánchez-Guirales S.A., Simon J.A., Tomietto G. (2024). Artificial Intelligence (AI) Applications in Drug Discovery and Drug Delivery: Revolutionizing Personalized Medicine. Pharmaceutics.

[17] Arden N.S., Fisher A.C., Tyner K., Yu L.X., Lee S.L., Kopcha M. (2021). Industry 4.0 for pharmaceutical manufacturing: Preparing for the smart factories of the future. Int. J. Pharm..

[18] Hole G., Hole A.S., McFalone-Shaw I. (2021). Digitalization in pharmaceutical industry: What to focus on under the digital implementation process?. Int. J. Pharm..

[19] Kaitin K.I. (2010). The Landscape for Pharmaceutical Innovation: Drivers of Cost-Effective Clinical Research. Pharm. Outsourc..

[20] DiMasi J.A., Grabowski H.G., Hansen R.W. (2016). Innovation in the pharmaceutical industry: New estimates of R&D costs. J. Health Econ..

[21] Wouters O.J., McKee M., Luyten J. (2020). Estimated Research and Development Investment Needed to Bring a New Medicine to Market, 2009–2018. JAMA.

[22] Schuhmacher A. (2024). Pharma innovation: How evolutionary economics is shaping the future of pharma R&D. Drug Discov. Today.

[23] Roope L.S.J. (2022). The economic challenges of new drug development. J. Control Release.

[24] Jaiswal R.K., Rastogi D., Vohora D., Singh G.S. (2018). Generic Drug and Bioequivalence Studies. Pharmaceutical Medicine and Translational Clinical Research.

[25] Raman R., Kowalski R., Achuthan K., Iyer A., Nedungadi P. (2025). Navigating artificial general intelligence development: Societal, technological, ethical, and brain-inspired pathways. Sci. Rep..

[26] Sarker I.H. (2022). AI-Based Modeling: Techniques, Applications and Research Issues Towards Automation, Intelligent and Smart Systems. SN Comput. Sci..

[27] Goodfellow I., Bengio Y., Courville A. (2016). Deep Learning.

[28] Heaton J., Goodfellow I., Bengio Y., Courville A. (2018). Deep learning. Genet. Program. Evolvable Mach..

[29] Ortiz B.L., Gupta V., Kumar R., Jalin A., Cao X., Ziegenbein C., Singhal A., Tewari M., Choi S.W. (2024). Data Preprocessing Techniques for AI and Machine Learning Readiness: Scoping Review of Wearable Sensor Data in Cancer Care. JMIR Mhealth Uhealth.

[30] Zhong H., Liu Y., Sun H., Liu Y., Zhang R., Li B., Yang Y., Huang Y., Yang F., Mak F.S. (2025). Towards global reaction feasibility and robustness prediction with high throughput data and bayesian deep learning. Nat. Commun..

[31] Zhang Y., Khan S.A., Mahmud A., Yang H., Lavin A., Levin M., Frey J., Dunnmon J., Evans J., Bundy A. (2025). Exploring the role of large language models in the scientific method: From hypothesis to discovery. npj Artif. Intell..

[32] Colliot O., Colliot O. (2023). A Non-technical Introduction to Machine Learning. Machine Learning for Brain Disorders.

[33] Mitchell T.M. (1997). Machine Learning.

[34] Du K.-L., Zhang R., Jiang B., Zeng J., Lu J. (2025). Understanding Machine Learning Principles: Learning, Inference, Generalization, and Computational Learning Theory. Mathematics.

[35] Hastie T., Tibshirani R., Friedman J. (2009). The Elements of Statistical Learning: Data Mining, Inference, and Prediction.

[36] Fang C., Wang Y., Grater R., Kapadnis S., Black C., Trapa P., Sciabola S. (2023). Prospective Validation of Machine Learning Algorithms for Absorption, Distribution, Metabolism, and Excretion Prediction: An Industrial Perspective. J. Chem. Inf. Model.

[37] Mater A.C., Coote M.L. (2019). Deep Learning in Chemistry. J. Chem. Inf. Model.

[38] Sun H., Burton H.V., Huang H. (2021). Machine learning applications for building structural design and performance assessment: State-of-the-art review. J. Build. Eng..

[39] Yu H., Mu Q., Liu C., Wang S., Sun J. (2025). Technical system of electroencephalography-based brain-computer interface: Advances, applications, and challenges. Neural Regen Res..

[40] Han J., Pei J., Kamber M. (2011). Data Mining: Concepts and Techniques.

[41] Bishop C.M. (2006). Pattern Recognition and Machine Learning.

[42] Janiesch C., Zschech P., Heinrich K. (2021). Machine learning and deep learning. Electron. Mark..

[43] Rosenblatt F. (1958). The perceptron: A probabilistic model for information storage and organization in the brain. Psychol. Rev..

[44] Café de Miranda G., de Lima G.G., Farias T.S. (2025). An introduction to neural networks for physicists. arXiv.

[45] Glorot X., Bengio Y. (2010). Understanding the difficulty of training deep feedforward neural networks. Proceedings of the Thirteenth International Conference on Artificial Intelligence and Statistics, Chia Laguna Resort, Italy, 13–15 May 2010.

[46] Rumelhart D., Hinton G., Williams R. (1986). Learning representations by back-propagating errors. Nature.

[47] Melody Y.K., Bidgoli H. (2003). Neural Networks. Encyclopedia of Information Systems.

[48] Ahmed S.F., Alam M.S.B., Kabir M., Afrin S., Rafa S.J., Mehjabin A., Gandomi A.H. (2025). Unveiling the frontiers of deep learning: Innovations shaping diverse domains. Appl. Intell..

[49] Russell S., Norvig P. (2020). Artificial Intelligence: A Modern Approach.

[50] Sarker I.H. (2021). Deep Learning: A Comprehensive Overview on Techniques, Taxonomy, Applications and Research Directions. SN Comput Sci..

[51] LeCun Y., Bengio Y., Hinton G. (2015). Deep learning. Nature.

[52] Wieder O., Kohlbacher S., Kuenemann M., Garon A., Ducrot P., Seidel T., Langer T. (2020). A compact review of molecular property prediction with graph neural networks. Drug Discov. Today Technol..

[53] Jumper J., Evans R., Pritzel A., Green T., Figurnov M., Ronneberger O., Tunyasuvunakool K., Bates R., Žídek A., Potapenko A. (2021). Highly accurate protein structure prediction with AlphaFold. Nature.

[54] Strobel G., Möller F., Banh L., Schoormann T. (2024). Exploring the taxonomy of generative AI. Res. Inf. Syst..

[55] Chithrananda S., Grand G., Ramsundar B. (2020). ChemBERTa: Large-scale self-supervised pretraining for molecular property prediction. arXiv.

[56] Watson J.L., Juergens D., Bennett N.R., Trippe B.L., Yim J., Eisenach H.E., Ahern W., Borst A.J., Ragotte R.J., Milles L.F. (2023). De novo design of protein structure and function with RFdiffusion. Nature.

[57] Rossi L.F., Sanna A., Manuri F., Bianco M.D. (2025). How AI-based computer vision algorithms are impacting the sports industry: A survey. Discov. Artif. Intell..

[58] Khadidos A.O., Yafoz A. (2025). An intelligent object detection and classification framework for assisting visually challenged persons using deep learning and improved crow search optimization. Sci. Rep..

[59] Wang B., Lei T., Cui B., Zhang B., Meng H., Nandi A.K. (2022). Medical image segmentation using deep learning: A survey. IET Image Process..

[60] Li G., Li K., Zhang G., Zhu Z., Wang P., Wang Z., Fu C. (2024). Enhanced multi view 3D reconstruction with improved MVSNet. Sci. Rep..

[61] Coelho C., Costa M.F.P., Ferrás L.L. (2024). Fractional Calculus Meets Neural Networks for Computer Vision: A Survey. AI.

[62] Mah P.M., Skalna I., Muzam J. (2022). Natural Language Processing and Artificial Intelligence for Enterprise Management in the Era of Industry 4.0. Appl. Sci..

[63] Otter D.W., Medina J.R., Kalita J.K. (2021). A Survey of the Usages of Deep Learning for Natural Language Processing. IEEE Trans. Neural Netw. Learn. Syst..

[64] Zadeh L.A. (1965). Fuzzy sets. Inf. Control..

[65] Klir G.J., Yuan B. (1995). Fuzzy Sets and Fuzzy Logic. Theory and Applications.

[66] Hansch C., Fujita T. (1964). ρ-σ-π analysis: A method for the correlation of biological activity and chemical structure. J. Am. Chem. Soc..

[67] Lindsay R.K., Buchanan B.G., Feigenbaum E.A., Lederberg J. (1993). DENDRAL: A case study of the first expert system for scientific hypothesis formation. Artif. Intell..

[68] Lavecchia A., Lavecchia A. (2026). History of artificial intelligence and drug discovery. Applied Artificial Intelligence for Drug Discovery.

[69] Bender A., Cortés-Ciriano I. (2021). Artificial intelligence in drug discovery: What is realistic, what are illusions? Part 1: Ways to make an impact, and why we are not there yet. Drug Discov. Today.

[70] Macarron R., Banks M.N., Bojanic D., Burns D.J., Cirovic D.A., Garyantes T., Green D.V., Hertzberg R.P., Janzen W.P., Paslay J.W. (2011). Impact of high-throughput screening in biomedical research. Nat. Rev. Drug Discov..

[71] Lipinski C.A., Lombardo F., Dominy B.W., Feeney P.J. (2001). Experimental and computational approaches to estimate solubility and permeability in drug discovery and development settings. Adv. Drug Deliv. Rev..

[72] Gaudelet T., Day B., Jamasb A.R., Soman J., Regep C., Liu G., Hayter J.B.R., Vickers R., Roberts C., Tang J. (2021). Utilizing graph machine learning within drug discovery and development. Brief. Bioinform..

[73] Hood L., Rowen L. (2013). The Human Genome Project: Big science transforms biology and medicine. Genome Med..

[74] McDonagh E.M., Trynka G., McCarthy M., Holzinger E.R., Khader S., Nakic N., Hu X., Cornu H., Dunham I., Hulcoop D. (2024). Human Genetics and Genomics for Drug Target Identification and Prioritization: Open Targets’ Perspective. Annu. Rev. Biomed. Data Sci..

[75] Sliwoski G., Kothiwale S., Meiler J., Lowe E.W. (2013). Computational methods in drug discovery. Pharmacol. Rev..

[76] Forli S., Huey R., Pique M.E., Sanner M.F., Goodsell D.S., Olson A.J. (2016). Computational protein-ligand docking and virtual drug screening with the AutoDock suite. Nat. Protoc..

[77] Cerqueira N.M., Gesto D., Oliveira E.F., Santos-Martins D., Brás N.F., Sousa S.F., Fernandes P.A., Ramos M.J. (2015). Receptor-based virtual screening protocol for drug discovery. Arch. Biochem. Biophys..

[78] Hopkins A.L. (2008). Network pharmacology: The next paradigm in drug discovery. Nat. Chem. Biol..

[79] Ma J., Sheridan R.P., Liaw A., Dahl G.E., Svetnik V. (2015). Deep neural nets as a method for quantitative structure-activity relationships. J. Chem. Inf. Model.

[80] Sarkar C., Das B., Rawat V.S., Wahlang J.B., Nongpiur A., Tiewsoh I., Lyngdoh N.M., Das D., Bidarolli M., Sony H.T. (2023). Artificial Intelligence and Machine Learning Technology Driven Modern Drug Discovery and Development. Int. J. Mol. Sci..

[81] Dahl G.E., Jaitly N., Salakhutdinov R. (2014). Multi-task neural networks for QSAR predictions. arXiv.

[82] Zhavoronkov A., Ivanenkov Y.A., Aliper A., Veselov M.S., Aladinskiy V.A., Aladinskaya A.V., Terentiev V.A., Polykovskiy D.A., Kuznetsov M.D., Asadulaev A. (2019). Deep learning enables rapid identification of potent DDR1 kinase inhibitors. Nat. Biotechnol..

[83] Zhou Y., Zhang Y., Zhang Z., Zhou Z., Zhu F. (2024). AI comes to the Nobel Prize and drug discovery. J. Pharm. Anal..

[84] Longhi S., Ventura S., Macedo-Ribeiro S., Radusky L.G., Kovačević J., Parra R.G., Andrade-Navarro M.A., Kajava A.V., Bednáriková Z., Monzon A. (2025). When artificial intelligence meets protein research. Open Res. Eur..

[85] Li B., Gilbert S. (2024). Artificial Intelligence awarded two Nobel Prizes for innovations that will shape the future of medicine. npj Digit. Med..

[86] Optic Inc., Mechanica Partners (2025). Expand Strategic Collaboration to Power AI-Driven Venture Creation Across Multiple Programs. BioSpace. https://www.biospace.com/press-releases/optic-and-mechanica-partners-expand-strategic-collaboration-to-power-ai-driven-venture-creation-across-multiple-programs.

[87] BiOptic. Technology https://bioptic.io/technology.

[88] Pantanowitz L., Hanna M., Pantanowitz J., Lennerz J., Henricks W.H., Shen P., Quinn B., Bennet S., Rashidi H.H. (2024). Regulatory Aspects of Artificial Intelligence and Machine Learning. Mod. Pathol..

[89] (2013). International Medical Device Regulators Forum (IMDRF) Software as a Medical Device Working Group Software as a Medical Device (SaMD): Key Definitions. https://www.imdrf.org/documents/software-medical-device-samd-key-definitions.

[90] Mermelstein H.T., Wallack J.J. (2008). Confidentiality in the age of HIPAA: A challenge for psychosomatic medicine. Psychosomatics.

[91] Edemekong P.F., Annamaraju P., Afzal M., Haydel M.J. (2025). Health Insurance Portability and Accountability Act (HIPAA) Compliance. StatPearls [Internet].

[92] US Food and Drug Administration (2023). Artificial Intelligence and Machine Learning (AI/ML) Software as a Medical Device (SaMD) Action Plan. https://www.fda.gov/media/145022/download.

[93] (2023). Executive Order on the Safe, Secure, and Trustworthy Development and Use of Artificial Intelligence. White House. https://www.govinfo.gov/content/pkg/FR-2023-11-01/pdf/2023-24283.pdf.

[94] Hickman E., Petrin M. (2021). Trustworthy AI and Corporate Governance: The EU’s Ethics Guidelines for Trustworthy Artificial Intelligence from a Company Law Perspective. Eur. Bus. Org. Law. Rev..

[95] van Kolfschooten H., van Oirschot J. (2024). The EU Artificial Intelligence Act (2024): Implications for healthcare. Health Policy.

[96] Busch F., Kather J.N., Johner C., Moser M., Truhn D., Adams L.C., Bressem K.K. (2024). Navigating the European Union Artificial Intelligence Act for Healthcare. npj Digit. Med..

[97] Laux J., Wachter S., Mittelstadt B. (2024). Trustworthy artificial intelligence and the European Union AI act: On the conflation of trustworthiness and acceptability of risk. Regul. Gov..

[98] Schliess F., Affini Dicenzo T., Gaus N., Bourez J.M., Stegbauer C., Szecsenyi J., Jacobsen M., Müller-Wieland D., Kulzer B., Heinemann L. (2024). The German Fast Track Toward Reimbursement of Digital Health Applications: Opportunities and Challenges for Manufacturers, Healthcare Providers, and People With Diabetes. J. Diabetes Sci. Technol..

[99] Cuggia M., Combes S. (2019). The French Health Data Hub and the German Medical Informatics Initiatives: Two National Projects to Promote Data Sharing in Healthcare. Yearb. Med. Inform..

[100] Ionașcu C.M. (2025). Artificial Intelligence Adoption in the European Union: A Data-Driven Cluster Analysis (2021–2024). Economies.

[101] Cojocaru A. (2025). Governing AI with trust: An adaptive framework for institutional legitimacy in the UK public sector. Transform. Gov. People Process Policy.

[102] Jia J., Zhao S. (2025). Ethical and Legal Governance of Generative AI in Chinese Healthcare. J. Multidiscip. Healthc..

[103] Wu W., Huang T., Gong K. (2020). Ethical Principles and Governance Technology Development of AI in China. Engineering.

[104] Migliorini S. (2024). China’s Interim Measures on generative AI: Origin, content and significance. Comput. Law Secur. Rev..

[105] Imai Y., Akatsu E., Minton S.K. (2025). Mitigate Japan’s Drug Loss with Model-Informed Drug Development. CPT Pharmacomet. Syst. Pharmacol..

[106] PMDA (2025). Regulatory Framework for Innovative Medical Devices. https://www.pmda.go.jp/files/000266836.pdf.

[107] The American Chamber of Commerce in Japan (2023). Charting a Path for Sustainable Healthcare in Japan. Health Policy White Paper.

[108] Brown D. Canada’s Proposed Artificial Intelligence and Data Act (AIDA): A Critical Review. https://ssrn.com/abstract=4687995.

[109] Zemlyannikova A.V. (2025). Experimental Legal Regimes as a Mechanism for Implementing Digital Innovations. Cour. Kutafin Mosc. State Law Univ..

[110] Murashko M.A., Vankov V.V., Panin A.I., Artemova O.R., Matvienko A.V., Gusev A.V., Vasilev Y.u.A., Vladzymyrskyy A.V. (2025). Implementation of artifi cial intelligence technologies in healthcare in Russia: Results of 2024. Natl. Health Care..

[111] Ministry of Digital Development, Innovations and Aerospace Industry of the Republic of Kazakhstan Digitalisation. https://www.gov.kz/article/201487?lang=en.

[112] Fialho de Oliveira R., Hering I., de Jesus T.C. (2025). AI and Digital Health in Brazil: Balancing Innovation and Regulation. International Bar Association. https://www.ibanet.org/ai-digital-health-brazil.

[113] Campos Ríos M. (2025). Artificial Intelligence and Public Policy in Latin America and the Caribbean: Experiences and Contributions Toward Shaping a Regional Roadmap. Caracas, Venezuela: Latin American and Caribbean Economic System (SELA).

[114] Nemko Digital AI Regulation in South Africa. Nemko Digital. https://digital.nemko.com/regulations/ai-regulation-in-south-africa.

[115] Lenarczyk G., Minssen T., Price N., Rai A. (2025). The future of AI regulation in drug development: A comparative analysis. J. Law Biosci..

[116] Niazi S.K. (2025). Regulatory Perspectives for AI/ML Implementation in Pharmaceutical GMP Environments. Pharmaceuticals.

[117] Roumeliotis K.I., Tselikas N.D. (2023). ChatGPT and Open-AI Models: A Preliminary Review. Future Internet.

[118] Genovese A., Hegstrom L., Prabha S., Gomez-Cabello C.A., Haider S.A., Collaco B., Wood N.G., Forte A.J. (2026). Artificial Authority: The Promise and Perils of LLM Judges in Healthcare. Bioengineering.

[119] Mullard A. (2016). Parsing clinical success rates. Nat. Rev. Drug Discov..

[120] Amorim A.M.B., Piochi L.F., Gaspar A.T., Preto A.J., Rosário-Ferreira N., Moreira I.S. (2024). Advancing Drug Safety in Drug Development: Bridging Computational Predictions for Enhanced Toxicity Prediction. Chem. Res. Toxicol..

[121] Enamine REAL Compounds, 2020. https://enamine.net/library-synthesis/real-compounds.

[122] Wishart D.S., Knox C., Guo A.C., Shrivastava S., Hassanali M., Stothard P., Chang Z., Woolsey J. (2006). DrugBank: A comprehensive resource for in silico drug discovery and exploration. Nucleic Acids Res..

[123] Xie X.Q. (2010). Exploiting PubChem for Virtual Screening. Expert. Opin. Drug Discov..

[124] Hastings J., Glauer M., Memariani A., Neuhaus F., Mossakowski T. (2021). Learning chemistry: Exploring the suitability of machine learning for the task of structure-based chemical ontology classification. J. Cheminform..

[125] Liu T., Lin Y., Wen X., Jorissen R.N., Gilson M.K. (2007). BindingDB: A web-accessible database of experimentally determined protein-ligand binding affinities. Nucleic Acids Res..

[126] Jamasb Arian R., Viñas R., Ma E.J., Harris C., Huang K., Hall D., Lió P., Blundell T.L. (2022). Graphein—A Python Library for Geometric Deep Learning and Network Analysis on Biomolecular Structures and Interaction Networks.

[127] Pan D., Ji Y. (2023). Extensible Business Reporting Language Technology Adoption and Diffusion—A Tripartite Evolutionary Game Perspective. Systems.

[128] Vinogradov V., Nguyen K.T., Steshin S., Izmailov I., Doronichev A. (2025). BIOPTIC B1 Ultra-High-Throughput Virtual Screening System Discovers LRRK2 Ligands in Vast Chemical Space. J. Chem. Inf. Model.

[129] Doronichev A. (2025). Why Drug Discovery Takes 15 Years—And How AI Cuts It to 3.

[130] Zhang Y., Mastouri M., Zhang Y. (2024). Accelerating drug discovery, development, and clinical trials by artificial intelligence. Med.

[131] Mustafa Y.F. (2026). Artificial intelligence-driven discovery of coumarin-based therapeutics: Revolutionizing target identification and validation. Pharm. Sci. Adv..

[132] Cassidy J., Cassidy J., Taylor B. (2020). Applications of Machine Learning in Drug Discovery I: Target Discovery and Small Molecule Drug Design. Artificial Intelligence in Oncology Drug Discovery and Development.

[133] Wang Q., Sun B., Yi Y., Velkov T., Shen J., Dai C., Jiang H. (2025). Progress of AI-Driven Drug–Target Interaction Prediction and Lead Optimization. Int. J. Mol. Sci..

[134] Fu C., Chen Q. (2025). The future of pharmaceuticals: Artificial intelligence in drug discovery and development. J. Pharm. Anal..

[135] Ding H., Tian J., Yu W., Wilson D.I., Young B.R., Cui X., Xin X., Wang Z., Li W. (2023). The Application of Artificial Intelligence and Big Data in the Food Industry. Foods.

[136] Mohs R.C., Greig N.H. (2017). Drug discovery and development: Role of basic biological research. Alzheimers. Dement..

[137] Paul S.M., Mytelka D.S., Dunwiddie C.T., Persinger C.C., Munos B.H., Lindborg S.R., Schacht A.L. (2010). How to improve R&D productivity: The pharmaceutical industry’s grand challenge. Nat. Rev. Drug Discov..

[138] Yetgin A. (2025). Revolutionizing multi-omics analysis with artificial intelligence and data processing. Quant. Biol..

[139] Schulte-Sasse R., Budach S., Hnisz D., Marsico A. (2021). Integration of multiomics data with graph convolutional networks to identify new cancer genes and their associated molecular mechanisms. Nat. Mach. Intell..

[140] Peng W., Tang Q., Dai W., Chen T. (2022). Improving cancer driver gene identification using multi-task learning on graph convolutional network. Brief. Bioinform..

[141] Zhang K., Yang X., Wang Y., Yu Y., Huang N., Li G., Li X., Wu J.C., Yang S. (2025). Artificial intelligence in drug development. Nat. Med..

[142] Ahmad B., Ouahada K., Hamam H. (2026). Machine learning for drug-target interaction prediction: A comprehensive review of models, challenges, and computational strategies. Comput. Struct. Biotechnol. J..

[143] Francoeur P.G., Masuda T., Sunseri J., Jia A., Iovanisci R.B., Snyder I., Koes D.R. (2020). Three-Dimensional Convolutional Neural Networks and a Cross-Docked Data Set for Structure-Based Drug Design. J. Chem. Inf. Model..

[144] Talukder M.A., Kazi M., Alazab A. (2025). Predicting drug-target interactions using machine learning with improved data balancing and feature engineering. Sci. Rep..

[145] Liao Q., Zhang Y., Chu Y., Ding Y., Liu Z., Zhao X., Wang Y., Wan J., Ding Y., Tiwari P. (2025). Application of Artificial Intelligence in Drug-target Interactions Prediction: A Review. npj Biomed. Innov..

[146] Panahandeh F., Mansouri N. (2025). A comprehensive review of neural network-based approaches for drug-target interaction prediction. Mol Divers..

[147] Talo M., Bozdag S. (2025). Top-DTI: Integrating topological deep learning and large language models for drug-target interaction prediction. Bioinformatics.

[148] Thafar M.A., Olayan R.S., Ashoor H., Albaradei S., Bajic V.B., Gao X., Gojobori T., Essack M. (2020). DTiGEMS+: Drug-target interaction prediction using graph embedding, graph mining, and similarity-based techniques. J. Cheminform..

[149] Hussein M.A., Munirathinam G. (2026). Artificial Intelligence-Driven Natural Product Discovery for Cancer Metastasis and Chemoresistance: From Computational Prediction to Preclinical Validation. Cancers.

[150] Du B.-X., Xu Y., Yiu S.-M., Yu H., Shi J.-Y. (2023). ADMET Property Prediction via Multi-task Graph Learning under Adaptive Auxiliary Task Selection. iScience.

[151] Wei Y., Li S., Li Z., Wan Z., Lin J. (2022). Interpretable-ADMET: A web service for ADMET prediction and optimization based on deep neural representation. Bioinformatics.

[152] Feinberg E.N., Joshi E., Pande V.S., Cheng A.C. (2020). Improvement in ADMET Prediction with Multitask Deep Featurization. J. Med. Chem..

[153] Cáceres E.L., Tudor M., Cheng A.C. (2020). Deep learning approaches in predicting ADMET properties. Future Med. Chem..

[154] Chuang K.V., Gunsalus L.M., Keiser M.J. (2020). Learning Molecular Representations for Medicinal Chemistry. J. Med. Chem..

[155] Myung Y., de Sá A.G.C., Ascher D.B. (2024). Deep-PK: Deep learning for small molecule pharmacokinetic and toxicity prediction. Nucleic Acids Res..

[156] Mouchlis V.D., Afantitis A., Serra A., Fratello M., Papadiamantis A.G., Aidinis V., Lynch I., Greco D., Melagraki G. (2021). Advances in de Novo Drug Design: From Conventional to Machine Learning Methods. Int. J. Mol. Sci..

[157] Meyers J., Fabian B., Brown N. (2021). De novo molecular design and generative models. Drug Discov. Today.

[158] Sousa T., Correia J., Pereira V., Rocha M. (2021). Generative Deep Learning for Targeted Compound Design. J. Chem. Inf. Model..

[159] Mauri A., Bertola M. (2024). AlvaBuilder: A Software for De Novo Molecular Design. J. Chem. Inf. Model..

[160] Srinivasan S., Batra R., Chan H., Kamath G., Cherukara M.J., Sankaranarayanan S.K.R.S. (2021). Artificial Intelligence-Guided De Novo Molecular Design Targeting COVID-19. ACS Omega..

[161] Blaschke T., Engkvist O., Bajorath J., Che H. (2020). Memory-assisted reinforcement learning for diverse molecular de novo design. J. Cheminform..

[162] Lin E., Lin C.H., Lane H.Y. (2020). Relevant Applications of Generative Adversarial Networks in Drug Design and Discovery: Molecular De Novo Design, Dimensionality Reduction, and De Novo Peptide and Protein Design. Molecules.

[163] Atz K., Cotos L., Isert C., Håkansson M., Focht D., Hilleke M., Nippa D.F., Iff M., Ledergerber J., Schiebroek C.C.G. (2024). Prospective de novo drug design with deep interactome learning. Nat. Commun..

[164] Götz J., Jackl M.K., Jindakun C., Marziale A.N., André J., Gosling D.J., Springer C., Palmieri M., Reck M., Luneau A. (2023). High-throughput synthesis provides data for predicting molecular properties and reaction success. Sci. Adv..

[165] Schneider G. (2018). Automating drug discovery. Nat. Rev. Drug Discov..

[166] Nippa D.F., Atz K., Stenzhorn Y., Müller A.T., Tosstorff A., Benz J., Binch H., Bürkler M., Haider A., Heer D. (2025). Expediting hit-to-lead progression in drug discovery through reaction prediction and multi-dimensional optimization. Nat. Commun..

[167] Koirala M., Yan L., Mohamed Z., DiPaola M. (2025). AI-Integrated QSAR Modeling for Enhanced Drug Discovery: From Classical Approaches to Deep Learning and Structural Insight. Int. J. Mol. Sci..

[168] Doron G., Genway S., Roberts M., Jasti S. (2025). Generative AI: Driving productivity and scientific breakthroughs in pharmaceutical R&D. Drug Discov. Today.

[169] Pfizer Inc. (2022). Data and AI Are Helping to Get Medicines to Patients Faster. https://www.pfizer.com/sites/default/files/investors/financial_reports/annual_reports/2022/story/data-and-ai-are-helping-to-get-medicines-to-patients-faster/.

[170] Pfizer’s AI Drug Discovery Cuts Years Off Development Time by Virtasant. https://www.virtasant.com/ai-today/revolutionizing-healthcare-pfizers-ai-journey-to-drug-discovery-and-personalized-medicine-2.

[171] Driving Patient-Centric Innovation in Life Sciences Using Generative AI with Pfizer—AWS. https://aws.amazon.com/solutions/case-studies/pfizer-PACT-case-study/.

[172] Pfizer’s Digital Transformation Journey: From COVID Response to Full-Scale Generative AI. https://www.dhitglobal.org/pfizers-digital-transformation-journey-from-covid-response-to-full-scale-generative-ai/.

[173] Merck & Co., Inc. Merck and Mayo Clinic Announce New Research and Development Collaboration to Support AI-Enabled Drug Discovery and Precision Medicine. https://www.merck.com/news/merck-and-mayo-clinic-announce-new-research-and-development-collaboration-to-support-ai-enabled-drug-discovery-and-precision-medicine/.

[174] Merck KGaA Merck Launches First Ever AI Solution to Integrate Drug Discovery and Synthesis. https://www.merckgroup.com/en/news/aiddison-drug-discovery-software-05-12-2023.html.

[175] Press Release: Sanofi “all in” on Artificial Intelligence and Data Science to Speed Breakthroughs for Patients. https://www.sanofi.com/en/media-room/press-releases/2023/2023-06-13-12-00-00-2687072.

[176] The Sanofi Transformation: How 2000+ Executives Are Driving a Data-Driven Revolution. https://www.hec.edu/en/executive-education/news/sanofi-transformation-how-2000-executives-are-driving-data-driven-revolution.

[177] How French Pharma Giant Sanofi is Betting Big on AI—Labiotech. https://www.labiotech.eu/in-depth/sanofi-ai-deals/.

[178] Sanofi Invests $180 Million Equity in Owkin’s Artificial Intelligence and Federated Learning to Advance Oncology Pipeline. https://www.sanofi.com/en/media-room/press-releases/2021/2021-11-18-06-30-00-2336966.

[179] AstraZeneca Data Science and AI in R&D: Transforming How We Discover and Develop Medicines. https://www.astrazeneca.com/r-d/data-science-and-ai.html.

[180] AstraZeneca Gain Early Access to AI-Driven ‘Digital Twins’ Courtesy of Altis Collab. https://www.fiercebiotech.com/cro/altis-leads-push-digital-twins-use-trials-includes-astrazeneca-and-bayer.

[181] Bayer, AstraZeneca Racks Up AI Partners, Seeking To Stop Cancer at All Stages—BioSpace. https://www.biospace.com/deals/astrazeneca-racks-up-ai-partners-seeking-to-stop-cancer-at-all-stages.

[182] Vanderbilt University Medical Center Bayer, VUMC to Advance Treatments for Cardiovascular and Kidney Diseases. https://news.vumc.org/2026/01/20/bayer-vumc-to-advance-treatments-for-cardiovascular-and-kidney-diseases/.

[183] Bayer A.G. (2024). Artificial Intelligence in Pharmaceuticals: Transforming Drug Discovery and Development. https://www.bayer.com/en/pharma/artificial-intelligence.

[184] Steinmetz K.L., Spack E.G. (2009). The basics of preclinical drug development for neurodegenerative disease indications. BMC Neurol..

[185] Eurasian Economic Commission (2019). Decision of the Board of the Eurasian Economic Commission No. 202 of 26 November 2019, on Approval of the Guidance on Non-clinical Safety Studies for the Conduct of Human Clinical Trials and Registration of Pharmaceuticals. Eurasian Economic Commission. https://www.consultant.ru/cons/cgi/online.cgi?req=doc&base=LAW&n=429232&dst=100011#gzNujCVz43M2Go69.

[186] Chen J., Hu Y., Cai M., Lu Y., Wang Y., Cao X., Lin M., Xu H., Wu J., Cao X. (2025). TrialBench: Multi-Modal AI-Ready Datasets for Clinical Trial Prediction. Sci. Data..

[187] Olguín V., Durán A., Las Heras M., Rubilar J.C., Cubillos F.A., Olguín P., Klein A.D. (2022). Genetic Background Matters: Population-Based Studies in Model Organisms for Translational Research. Int. J. Mol. Sci..

[188] Nagy A., Perrimon N., Sandmeyer S., Plasterk R. (2003). Tailoring the genome: The power of genetic approaches. Nat. Genet..

[189] Gordon-Larsen P., French J.E., Moustaid-Moussa N., Voruganti V.S., Mayer-Davis E.J., Bizon C.A., Cheng Z., Stewart D.A., Easterbrook J.W., Shaikh S.R. (2021). Synergizing Mouse and Human Studies to Understand the Heterogeneity of Obesity. Adv. Nutr..

[190] Olson H., Betton G., Robinson D., Thomas K., Monro A., Kolaja G., Lilly P., Sanders J., Sipes G., Bracken W. (2000). Concordance of the toxicity of pharmaceuticals in humans and in animals. Regul. Toxicol. Pharmacol..

[191] Liu X., Fan F. (2026). A large-scale concordance study of toxicity findings across preclinical species and humans for small molecules and biologics. Front Toxicol..

[192] Tosca E.M., Ronchi D., Facciolo D., Magni P. (2023). Replacement, Reduction, and Refinement of Animal Experiments in Anticancer Drug Development: The Contribution of 3D In Vitro Cancer Models in the Drug Efficacy Assessment. Biomedicines.

[193] Kaplan B.L.F., Hoberman A.M., Slikker W., Smith M.A., Corsini E., Knudsen T.B., Marty M.S., Sobrian S.K., Fitzpatrick S.C., Ratner M.H. (2024). Protecting Human and Animal Health: The Road from Animal Models to New Approach Methods. Pharmacol. Rev..

[194] Opportunities CRACK IT Mega-Challenge: Towards a Virtual Second Species in Toxicology for Drug Development. https://iuk-business-connect.org.uk/opportunities/crack-it-mega-challenge-towards-a-virtual-second-species-in-toxicology-for-drug-development/.

[195] esqLABS esqLABS Awarded £1.6M to Develop a Virtual Second Species. https://esqlabs.com.

[196] Ineichen B.V., Furrer E., Grüninger S.L., Zürrer W.E., Macleod M.R. (2024). Analysis of animal-to-human translation shows that only 5% of animal-tested therapeutic interventions obtain regulatory approval for human applications. PLoS Biol..

[197] Howells D.W., Sena E.S., Macleod M.R. (2014). Bringing rigour to translational medicine. Nat. Rev. Neurol..

[198] Ferreira F.J.N., Carneiro A.S. (2025). AI-Driven Drug Discovery: A Comprehensive Review. ACS Omega.

[199] Ouedraogo G., Alépée N., Tan B., Roper C.S. (2025). A call to action: Advancing new approach methodologies (NAMs) in regulatory toxicology through a unified framework for validation and acceptance. Regul. Toxicol. Pharmacol..

[200] Silva M.C., Haggarty S.J. (2020). Human pluripotent stem cell-derived models and drug screening in CNS precision medicine. Ann. N. Y. Acad. Sci..

[201] Adams W.J., García-Cardeña G. (2012). Novel stem cell-based drug discovery platforms for cardiovascular disease. J. Biomol. Screen..

[202] Lee H., Kim J., Kim J.W., Lee Y. (2025). Recent advances in AI-based toxicity prediction for drug discovery. Front. Chem..

[203] Lu J., Wu L., Li R., Wan M., Yang J., Zan P., Bai H., He S., Bo X. (2025). ToxACoL: An endpoint-aware and task-focused compound representation learning paradigm for acute toxicity assessment. Nat. Commun..

[204] Paik D.T., Chandy M., Wu J.C. (2020). Patient and Disease-Specific Induced Pluripotent Stem Cells for Discovery of Personalized Cardiovascular Drugs and Therapeutics. Pharmacol. Rev..

[205] Chen X., Bao F., Liu J., Wang Y., Tao T., Zhang G., Qin J. (2026). A human liver organoids-on-chip for the assessment of drug-induced liver injury. BMC Pharmacol. Toxicol..

[206] Ren M., Feng Q., Lu S., Xiao W., Deng Q., Yang X., Yan C., Ren Z., Yan B., Wu Y. (2026). Application of HepG2-derived organoid and organ-on-a-chip platforms in elucidating selenium-mediated protection against cadmium-induced liver dysfunction. npj Sci. Food.

[207] Weaver D.T., King E.S., Maltas J., Scott J.G. (2024). Reinforcement Learning informs optimal treatment strategies to limit antibiotic resistance. Proc. Natl. Acad. Sci. USA.

[208] Jayaraman P., Desman J., Sabounchi M., Nadkarni G.N., Sakhuja A. (2024). A Primer on Reinforcement Learning in Medicine for Clinicians. npj Digit. Med..

[209] van den Broek R.L., Patel S., van Westen G.J.P., Jespers W., Sherman W. (2025). In Search of Beautiful Molecules: A Perspective on Generative Modeling for Drug Design. J. Chem. Inf. Model.

[210] Chen X., Roberts R., Tong W., Liu Z. (2022). Tox-GAN: An Artificial Intelligence Approach Al-ternative to Animal Studies-A Case Study with Toxicogenomics. Toxicol. Sci..

[211] Dai M., Xiao G., Shao M., Zhang Y.S. (2023). The Synergy between Deep Learning and Or-gans-on-Chips for High-Throughput Drug Screening: A Review. Biosensors.

[212] Bai L., Wu Y., Li G., Zhang W., Zhang H., Su J. (2023). AI-enabled organoids: Construction, analy-sis, and application. Bioact. Mater..

[213] Ferrari E., Rasponi M. (2021). Liver-Heart on chip models for drug safety. APL Bioeng..

[214] Simoens S., Huys I. (2021). R&D Costs of New Medicines: A Landscape Analysis. Front. Med..

[215] European Medicines Agency (2025). ICH E6 (R3) Guideline for Good Clinical Practice (GCP). https://www.ema.europa.eu/en/documents/scientific-guideline/ich-e6-r3-guideline-good-clinical-practice-gcp-step-5_en.pdf.

[216] Berry D.A. (2025). Adaptive Bayesian Clinical Trials: The Past, Present, and Future of Clinical Research. J. Clin. Med..

[217] Pallmann P., Bedding A.W., Choodari-Oskooei B., Dimairo M., Flight L., Hampson L.V., Holmes J., Mander A.P., Odondi L., Sydes M.R. (2018). Adaptive designs in clinical trials: Why use them, and how to run and report them. BMC Med..

[218] Goodyear M.D., Krleza-Jeric K., Lemmens T. (2007). The Declaration of Helsinki. BMJ.

[219] World Medical Association Ethics Unit. Declaration of Helsinki 2007. https://www.wma.net/policies-post/wma-declaration-of-helsinki/.

[220] (2013). IMDRF SaMD Working Group Software as a Medical Device (SaMD): Key Definitions. International Medical Device Regulators Forum (IMDRF). https://www.imdrf.org/sites/default/files/docs/imdrf/final/technical/imdrf-tech-131209-samd-key-definitions-140901.pdf.

[221] Ebad S.A., Alhashmi A., Amara M., Miled A.B., Saqib M. (2025). Artificial Intelligence-Based Software as a Medical Device (AI-SaMD): A Systematic Review. Healthcare.

[222] Pannonhalmi Á., Sipos B., Kurucz R.I., Katona G., Kemény L., Csóka I. (2025). Advancing Regulatory Oversight of Medical Device Trials to Align with Clinical Drug Standards in the European Union. Pharmaceuticals.

[223] Gupte T., Nitave T., Gobburu J. (2025). Regulatory landscape of accelerated approval pathways for medical devices in the United States and the European Union. Front. Med. Technol..

[224] Vaterkowski M., Daniel C., La Rosa A., Kalra D., Jaulent M.C., Lame G., Kempf E. (2025). Electronic Health Record based recruitment support systems: A scoping review to improve patient inclusion within clinical trials. Int. J. Med. Inform..

[225] Yang Y., Yuan Y., Zhang G., Wang H., Chen Y.C., Liu Y., Tarolli C.G., Crepeau D., Bukartyk J., Junna M.R. (2022). Artificial intelligence-enabled detection and assessment of Parkinson’s disease using nocturnal breathing signals. Nat. Med..

[226] Leveraging Synthetic Control Arms in Clinical Trials and RWE Studies. IQVIA. https://www.iqvia.com/locations/middle-east-and-africa/library/white-papers/leveraging-synthetic-control-arms-in-clinical-trials-and-rwe-studies.

[227] Mørk S.K., Kadivar M., Bol K.F., Draghi A., Westergaard M.C.W., Skadborg S.K., Overgaard N., Sørensen A.B., Rasmussen I.S., Andreasen L.V. (2022). Personalized therapy with peptide-based neoantigen vaccine (EVX-01) including a novel adjuvant, CAF^®^09b, in patients with metastatic melanoma. Oncoimmunology.

[228] Long G.V., Ferrucci P.F., Khattak A., Meniawy T.M., Ott P.A., Chisamore M., Trolle T., Agon H., Heegaard E. (2022). KEYNOTE—D36: Personalized immunotherapy with a neoepitope vaccine, EVX-01 and pembrolizumab in advanced melanoma. Future Oncol..

[229] Dermawan D., Alotaiq N. (2025). From Lab to Clinic: How Artificial Intelligence (AI) Is Reshaping Drug Discovery Timelines and Industry Outcomes. Pharmaceuticals.

[230] Tikhomirov L., Semmler C., Prizant N., Bhasin S., Kenyon G., van der Vegt A., Erdman L., Kurian N.C., Thompson H., Palmer L.J. (2026). A scoping review of silent trials for medical artificial intelligence. Nat. Health.

[231] Ringold D., Weitz B. (2007). The American Marketing Association Definition of Marketing: Moving from Lagging to Leading Indicator. J. Public Policy Mark..

[232] Amin M.R.M., Asbi A., Sivakumaran V.M., Kim J., Septiarini E. (2025). Artificial Intelligence (AI) adoption in marketing strategies: Navigating the present and shaping the future business landscape. Soc. Sci. Humanit. Open..

[233] Huang M.H., Rust R.T. (2021). A strategic framework for artificial intelligence in marketing. J. Acad. Mark. Sci..

[234] Eve Nova (2025). Integrating Big Data and Artificial Intelligence for Dynamic Market Segmentation and Product Positioning.

[235] Agarwal R., Dugas M., Gao G., Kannan P.K. (2020). Emerging technologies and analytics for a new era of value-centered marketing in healthcare. J. Acad. Mark. Sci..

[236] Fourkiotis K.P., Tsadiras A. (2024). Applying Machine Learning and Statistical Forecasting Methods for Enhancing Pharmaceutical Sales Predictions. Forecasting.

[237] Precedence Research (2024). AI in Pharmaceutical Market—Global Industry Analysis and Forecast. https://www.precedenceresearch.com/ai-in-pharmaceutical-market.

[238] Coherent Solutions Artificial Intelligence in Pharmaceuticals and Biotechnology: Current Trends and Innovations. Published 2024. https://www.coherentsolutions.com/insights/artificial-intelligence-in-pharmaceuticals-and-biotechnology-current-trends-and-innovations.

[239] Zainab J., János D. (2025). A Comprehensive Review of AI-Enhanced Decision Making: An Empirical Analysis for Optimizing Medication Market Business. Mach. Learn. Appl..

[240] Ozay D., Jahanbakht M., Shoomal A., Wang S. (2024). Artificial Intelligence (AI)-based Customer Relationship Management (CRM): A comprehensive bibliometric and systematic literature review with outlook on future research. Enterp. Inf. Syst..

[241] Ruschemeier H., Steffen B. (2025). Thinking Outside the Box?. Bridging the Gap Between AI and Reality.

[242] Beetler D.J., Di Florio D.N., Law E.W., Groen C.M., Windebank A.J., Peterson Q.P., Fairweather D. (2023). The evolving regulatory landscape in regenerative medicine. Mol. Asp. Med..

[243] Klonoff D.C. (2020). The New FDA Real-World Evidence Program to Support Development of Drugs and Biologics. J. Diabetes Sci. Technol..

[244] Janiaud P., Irony T., Russek-Cohen E., Goodman S.N. (2021). U.S. Food and Drug Administration Reasoning in Approval Decisions When Efficacy Evidence Is Borderline, 2013–2018. Ann. Intern. Med..

[245] Harvey H.B., Gowda V. (2020). How the FDA Regulates AI. Acad. Radiol..

[246] Pasas-Farmer S., Rashi J. (2025). From discovery to delivery: Governance of AI in the pharmaceutical industry. Green Anal. Chem..

[247] Xu H., Shuttleworth K. (2024). Medical artificial intelligence and the black box problem: A view based on the ethical principle of “do no harm”. Intell. Med..

[248] Chan B. (2023). Black-box assisted medical decisions: AI power vs. ethical physician care. Med. Health Care Philos..

[249] Cross J.L., Choma M.A., Onofrey J.A. (2024). Bias in medical AI: Implications for clinical decision-making. PLoS Digit. Health.

[250] Chen F., Wang L., Hong J., Jiang J., Zhou L. (2024). Unmasking bias in artificial intelligence: A systematic review of bias detection and mitigation strategies in electronic health record-based models. J. Am. Med. Inform. Assoc..

[251] Martínez-García M., Hernández-Lemus E. (2022). Data Integration Challenges for Machine Learning in Precision Medicine. Front. Med..

[252] Sahu M., Gupta R., Ambasta R.K., Kumar P. (2022). Artificial intelligence and machine learning in precision medicine: A paradigm shift in big data analysis. Prog. Mol. Biol. Transl. Sci..

[253] Hassan M., Kushniruk A., Borycki E. (2024). Barriers to and Facilitators of Artificial Intelligence Adoption in Health Care: Scoping Review. JMIR Hum. Factors..

[254] Ponce T., Enrique M., Arana, Noel M. Artificial Intelligence in USA. Healthcare: Legal Framework, Liability, and Ethical Challenges with Special Focus on Pediatric Care. https://ssrn.com/abstract=5468846.

[255] Ocana A., Pandiella A., Privat C., Bravo I., Luengo-Oroz M., Amir E., Gyorffy B. (2025). Integrating artificial intelligence in drug discovery and early drug development: A transformative approach. Biomark. Res..

[256] Jämsä-Jounela S.-L. (2007). Future trends in process automation. Annu. Rev. Control.

[257] Davenport T.H., Ronanki R. (2018). Artificial intelligence for the real world. Harv. Bus. Rev..

[258] Wu S., Liu Y., Ruan M., Chen S., Xie X.Y. (2025). Human-generative AI collaboration enhances task performance but undermines human’s intrinsic motivation. Sci. Rep..

